# Combined Effect of Climate and Anthropopressure on River Water Quality

**DOI:** 10.3390/ijerph20043032

**Published:** 2023-02-09

**Authors:** Kinga Wieczorek, Anna Turek, Wojciech M. Wolf

**Affiliations:** Institute of General and Ecological Chemistry, Lodz University of Technology, 116 Żeromskiego Str., 90-924 Łódź, Poland

**Keywords:** river water, pollution, climate, anthropopressure, monitoring, water quality, multivariate statistics

## Abstract

This study was a continuation of our investigation of the spatio-temporal variability of the Bzura River’s water chemistry. Our research is of particular importance in the context of the recent ecological disaster on the Oder River and concerns the international problem of surface water contamination. The study area was a 120 km section of the Bzura River. We tested more measurement points and with a higher sampling frequency than those used in the national monitoring of river water quality. During two hydrological years, 360 water samples were collected. The selected parameters: electrical conductivity, temperature, dissolved oxygen, dissolved organic carbon, nitrates, phosphates, bicarbonates, chlorides, sodium, potassium, calcium, and magnesium were determined. Numerous results exceeded the Polish threshold limits. Spatio-temporal variability and water quality were assessed using principal component analysis (PCA), cluster analysis (CA), and water quality index (WQI) approaches. Many point sources of pollution related to urbanization, agriculture, and industry were detected. Moreover, due to the changing climatic conditions, a significant difference between temporal variability in both years was observed. Our results indicated that it is necessary to increase the number of measurement stations for surface water monitoring; it will allow for a faster detection of the threat.

## 1. Introduction

Rivers are a source of freshwater, which is crucial for human development [1]. River water quality strongly depends on anthropogenic factors and climate conditions. An increasing problem of drinking water deficiency requires the special protection of available water resources and careful monitoring of river water chemistry [2]. However, environmental monitoring should account for the current climate and land use changes [3].

In Poland, majority of renewable surface water resources come from precipitation. The volume of water per capita is only 1500 m^3^, and it is one of the lowest values in the European Union (EU) [4]. In addition, the water exploitation index (WEI, calculated as the proportion of the freshwater consumed compared with the amount of renewable freshwater resources) in 2017 was 17.7%. According to the European Environment Agency (EEA), values below 20% mean water deficiency [5]. In Poland, the state of surface waters is controlled by the state environmental monitoring (SEM) program, which was established in 1991. Until 2018, the SEM was run by the Voivodeship Inspectorates for Environmental Protection (VIEP), but after the amendment to the Act on the Inspectorate of Environmental Protection, it is now realized by the Chief Inspectorate for Environmental Protection (CIEP) [6]. This change influenced the way of presenting the results of monitoring. Until 2017, detailed analyses were published on the websites of VIEP; since 2018, only concise annual reports on the state of the environment have been published [7]. Unfortunately, access to detailed results is not straightforward. Currently, the tasks specified in the Strategic Program of the SEM for the years of 2020–2025 are being implemented; this document defines the number of measurement control points, the frequency of tests, and the scope of analyses [8].

The ecological disaster on the Oder River (the river that runs along the Polish–German border and flows into the Baltic Sea), which took place in August 2022 [9], revealed some weak points of the Polish river monitoring system. The river water was contaminated, which caused the death of 100 [10]–250 tons [11] (depending on the source) of fish on the Polish side and about 30 tons on the German side [12]. The river was contaminated by toxins produced by golden algae (*Prymnesium parvum*), which is not normally found in freshwater [10]. However, the discharge of brine waters into the Oder River (perhaps also an additional chemical spill) and the climate conditions (a high temperature and low water level additionally contributed to the increase in the concentration of pollutants) [11] led to the creation of an “effective bioreactor for the cultivation of brackish water algae” [9]. Based on the satellite data (analysis of chlorophyll concentration in the river), it can be seen that the first algae bloom already started at the end of July in the upper course of the river. At the beginning of August, there was a rapid increase in the concentration of chlorophyll, and then the mass of phytoplankton very quickly spread downstream and covered almost the entire river within a week [13]. There are only 10 measurement control points along the entire length of the Oder River (840 km). Since the beginning of August, water analyses have been carried out every few days [14], but before the disaster, they were carried out depending on the kind of monitoring and the type of indicator [15,16] from once to twelve times a year [17]. A greater number of points and frequency of analyses would probably allow for an earlier reaction from the relevant services and could significantly reduce the scale of a disaster [18].

Another important river in the catchment area of the Baltic Sea is the Bzura River. It is a left tributary of the largest Polish river, the Vistula. The Bzura River is subjected to strong anthropopressure. On the section of this river that is our research area (120 km within the boundaries of the District of Łódź), there are only four measurement control points of SEM, which are separated from each other by distances of 15 km, 58 km, and 24 km, respectively. In 2006 [19], the Bzura River water quality was monitored at eleven points, but since 2008 [20], inspections have been limited to only being carried out in the above-mentioned points [21]. Tests are performed once a month or at even longer intervals. Moreover, measurements are often not carried out due to the unfavorable weather conditions. For example, in 2018, not all planned studies were performed as a result of drought [21]. The scope of chemical analyses planned in the Strategic Program of the SEM is wide, but only some of them are monitored in practice. The number of parameters that must be analyzed varies depending on the type of monitoring and the value of a given parameter that is determined in previous years. In 2017, when we started our research, analyses were mainly carried out for contents of mercury and five selected polycyclic aromatic hydrocarbons [22]. In 2018, over 90 parameters were checked, whereas in 2019, only 5–9 indicators were tested. Apart from the research conducted as part of the SEM, Bzura is not well-known in terms of chemical indicators, because most of the scientific work concerns forest vegetation [23], fish fauna [24], or restoration [25]. There is still a lack of comprehensive analyses of the parameters that may (directly or indirectly) pose a risk of ecological disaster. Therefore, in 2018–2019 (two hydrological years), we performed systematic, monthly analyses of 21 physicochemical parameters of water quality in 13 (2018) and 17 (2019) sites located on the Bzura River. The research concerned the indicators covered by the SEM monitoring, i.e., temperature, pH, electrical conductivity (EC), total dissolved solids (TDS), dissolved oxygen (DO), dissolved organic carbon (DOC), bicarbonates, chlorides, nitrates, phosphates, calcium, magnesium, copper, zinc, cadmium, lead, and nickel, and additionally sodium, potassium, iron, and manganese. In our previous paper, we discussed the contamination of the Bzura water with heavy metals (Ni, Cd, Pb, Cu, Zn, Mn, and Fe) [26]. Apart from the last two elements, these heavy metals, even at low concentrations, may directly pose a threat to aquatic organisms; indirectly, they also pose a threat to terrestrial flora and fauna. Increased contents of Fe and Mn cause, e.g., oxygen deficit in the water. In the research, we also took into account pH (a factor determining the solubility of pollutants accumulated in the bottom sediments of rivers) and TDS (characterizing salinity). In the current study, we focused on the variability of the other parameters mentioned above. They are very important due to the use of surface water for food and industrial purposes. Temperature is crucial to oxygen content, the development of aquatic organisms, and the interactions between water components and bottom sediments. The variability of the dissolved oxygen (DO) concentration is caused by both natural factors and pollutants. Dissolved organic carbon (DOC) is commonly used in water quality monitoring to assess the content of organic substances, especially their non-biodegradable fractions. Electrical conductivity (EC) is an indicator of water contamination with soluble substances. Their sources are soil minerals, as well as substances introduced as a result of human activity. Bicarbonates have buffering properties and are responsible for the alkalinity of water (a favorable phenomenon when water is used for industrial purposes). Nitrates and phosphates in surface waters are indicators of pollution from agriculture and wastewater; together with potassium, they are a source of eutrophication in water reservoirs. The salinity of surface waters is caused by the presence of highly soluble compounds, with sodium and chloride ions having the largest share in salinity. The elements necessary for the development of organisms are calcium and magnesium. The sources of these elements are primarily from natural geological processes, but can also come from economic activity. 

The objectives of this work are: (i) to take measurements of selected physicochemical and chemical parameters in the Bzura River water; (ii) assess the pollution levels and identify pollutant sources; (iii) assess the impact of climate and human activity on the spatio-temporal variability of individual parameters of river water quality; and (iv) propose the optimal number of control points and suggest the frequency of monitoring tests.

## 2. Materials and Methods

### 2.1. Study Area

**Figure 1:**
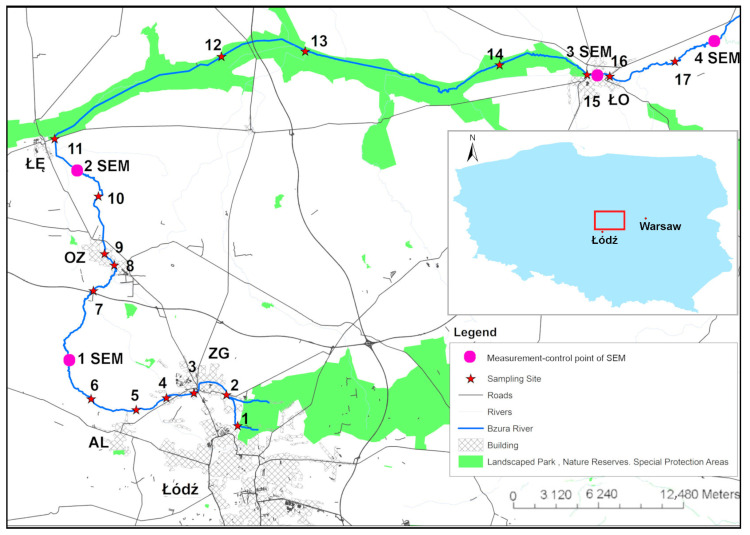
Sampling sites and measurement control points of the SEM location.

The Bzura River is a lowland river of medium length (165 km). It flows through the Central Poland Lowlands in the area between Łódź and Warsaw. The river has its sources on the outskirts of the city of Łódź (the fourth-largest city in Poland in terms of the number of inhabitants). In the examined section, the Bzura River flows through smaller towns: Zgierz (ZG), Aleksandrów Łódzki (AL), Ozorków (OZ), Łęczyca (ŁĘ), and Łowicz (ŁO)–Figure 1. A detailed characteristic of the study area is given in our previous research [26] and is presented in Table S1. Figure 1 shows the location of the measurement control points of the SEM relative to the sampling sites selected for our study.

### 2.2. Sampling, Preparation, and River Water Analysis

Sampling and preparation (filtration, acidification, and storage) were performed as described by Wieczorek et al. [26]; therefore, only the information that has not previously appeared will be presented. Water samples were taken from 13 and 17 sites in 2018 and 2019, respectively. A total of 360 samples were collected.

The EC was measured in situ by the conductometric method (EC-meter Series 410, Elmetron, Zabrze, Poland). The DO was measured in situ using a mobile oxygen meter (Serie CO-402, Elmetron, Zabrze, Poland). The measurements of temperature and DOC have been previously described [26]. The samples for the nitrates and phosphates concentration analysis were collected in a glass bottle to the top, acidified with 1 mL of sulfuric acid (96%, d = 1.84 g/mL), filtered, and stored in a refrigerator. The measurement was performed within 24 h. The water for the bicarbonates and chlorides analysis was collected in PE bottles to the top, filtered (0.45 µm pore size), and stored in a refrigerator (maximum storage time 24 h for HCO_3_ and one week for Cl). For the sodium, potassium, calcium, and magnesium determination, river water sampling and preparation were carried out in the same way as for the metals in our previous work. 

The NO_3_ concentration was measured by the potentiometric method using a pH/mV meter (Delta 350 Mettler Toledo, Columbus, OH, USA) equipped with a nitrate ion-selective electrode (Detektor, Raszyn, Poland) and a Ag/AgCl reference electrode (RL-100 type, Hydromet, Gliwice, Poland). The content of PO_4_ was determined by the spectrophotometric method (Spekol 11 spectrophotometer, Carl Zeiss, Jena, Germany) with a vanadate-molybdate reagent. The HCO_3_ content was determined by the volumetric method using an automatic titrator (Mettler Toledo, Greifensee, Switzerland). The Cl concentration was calculated based on an argentometric titration analysis (Mohr method). Atomic absorption spectrometry with flame atomization (FAAS) (ContrAA 300, Analytik, Jena, Germany) was applied for Ca and Mg determination. The concentrations of Na and K were measured by the photometric method with a flame photometer (Serie BWB-XP, BWB Technologies, Newbury, UK). Calibration curves were prepared by dilution of a standard solution of metals (Certipur, Merck Supelco, Darmstadt, Germany). The limits of detection (LODs) were determined based on the blank samples analysis (n = 20). The LODs were 0.03 mg/L (Ca), 0.001 mg/L (Mg), 0.18 mg/L (Na), and 0.07 mg/L (K).

For quality control and assurance, the environmental matrix reference material (RM) ION-96.4 (Environment and Climate Change Canada, Burlington, ON, Canada) was analyzed. This RM is a natural river water from Grand River (Ontario). Recoveries of 85.5% for K, 98.8% for Ca, 102% for Mg, and 104% for Na were obtained. Detailed results of the RM analysis are depicted in Table A1. Moreover, the analytical precision was verified by the analysis of eight parallel water samples collected at site 4. The relative standard deviations (RSDs) ranged from 1.8% to 5.4%.

The laboratory glassware was washed in 10% HNO_3_ for 24 h and rinsed with deionized water. Blank reagents (prepared in the same way as samples) were involved for each analytical procedure. Samples in triplicate were applied for all measurements. For chemical analyses, Certipur or ACS-grade reagents and deionized water (electrical conductivity of 0.05 µS) were used.

### 2.3. Data Development

The statistical parameters (mean, minimum, and maximum values) for each sampling site and each month in 2018 and 2019 were calculated. The Shapiro–Wilk (S-W) test was used for the distribution normality of the data assessment. Inter-annual spatio-temporal variability was separately investigated for each parameter based on the Pearson (normal distribution) or Spearman (non-normal distribution) correlation coefficients, coefficients of variation (CVs), and cluster analysis (CA). The CA was also performed for datasets that contained all parameters (based on annual or monthly mean values) and was supported by the principal component analysis (PCA). Before the PCA and CA analyses, column autoscaling was applied. Ward’s method and the squared Euclidean distance were used for CA calculations. Kaiser’s criterion and the cumulative percentage of the explained variance for the principal components (PCs) selection were implemented. Statistical calculations were executed with Statistica 10 software (StatSoft Inc., Tulsa, OK, USA). 

The water quality index (WQI) was used to assess the river water quality [27]:

WQI = Σ w_i_q_i_/Σ w_i_ (1)

where:

q_i_—Quality rating scale of the i^th^ parameter; q_i_ = (C_i_/S_i_)*100;

C_i_—Value of the ith parameter;

S_i_—Threshold value of the ith parameter;

w_i_—Unit weight of the ith parameter

Based on the WQI values, water can be classified as:

Excellent   WQI < 50

Good    50 ≤ WQI < 100

Poor     100 ≤ WQI < 200

Very poor  200 ≤ WQI < 300

Unsuitable  WQI ≥ 300

## 3. Results and Discussion

### 3.1. Spatio-Temporal Variations of Measured Parameters

The range and mean values of the parameters measured in the Bzura River water samples are presented in Table 1. 

Based on the mean values, the cation concentrations increased in the following order: K < Mg < Na < Ca (in 2018 for all points and in 2019 for points 14–17) and K ≈ Mg < Ca < Na (in 2019 in the other sites). The order of anion concentrations was PO_4_ < NO_3_ < Cl < HCO_3_ in both years. Similar trends were observed for other Polish and European rivers [28,29,30]. 

Considering the river section consisting of 13 sampling points, the ranges of the parameters value differed depending on the hydrological year (except for NO_3_, PO_4_, EC, and water temperature). For DO, DOC, HCO_3_, K, Ca, and Mg, higher variability occurred in 2018. Only the Cl and Na concentrations were more diverse in 2019. The lower concentrations (except for NO_3_) were obtained in 2019 for the four additional sampling points of 14–17. 

According to Polish regulation [31], surface water quality can be classified in terms of physicochemical and chemical parameters as class I (very good quality) and class II (good quality). If the limit values for class II are not achieved, the water quality is determined as below good. In both 2018 and 2019, the threshold values (Table A2) for class II were not exceeded only in the case of temperature. Less than 50% of all results (sites 1–13) exceeded these limits for EC, DO, Mg, NO_3_, and HCO_3_ in 2018 and 2019, and also for Ca in 2019 (Table S2). In the case of the remaining parameters, more than 70% of the results exceeded threshold values for water class II.

#### 3.1.1. Inter-Annual Spatio-Temporal Variability

In order to compare the temporal variability of the Bzura River chemistry, the correlation coefficients for individual parameters at each sampling site (2018 vs. 2019) were computed (Table 2).

Most of the significant inter-annual correlations for temporal variability occurred for the water temperature and the DO parameters (Table 2). They were strongly positively correlated [32] at almost all sites. This indicates that these parameters were strongly linked to the seasons, and their fluctuation was similar in both years. Significantly negative correlation coefficients were obtained for the EC at sites 1, 11, and from 7 to 9, which suggests different temporal trends in both years. In the case of the remaining parameters, statistically significant correlations were only identified for a few sites, e.g., for the NO_3_ at sites 11 and 12, which is probably associated with the fertilization. Additionally, site 13 was characterized by moderate (≥0.5) and strong correlations for Cl, K, and Na. It may relate to the continuous supply of these ions by one of the Bzura River tributaries (the Ochnia River), which is heavily loaded with wastewater discharged from the industrial area; its estuary is located above site 13.

In order to compare the spatial variability of the Bzura River chemistry, the correlation coefficients of individual parameters for each month (2018 vs. 2019) were calculated (Table 3).

More significant correlations were obtained for the inter-annual spatial variability than for the inter-annual temporal variability (Table 3). All coefficients were positive, and moderate or strong correlations indicated that the spatial trends were more repetitive than the temporal ones. It may be linked to, e.g., the land use or the location of potential pollutant emitters, which were similar in both years. Strong correlations mainly existed for the EC as well as the Na, K, and Cl content in all months. These parameters probably depend more on the spatial system of the river than on the seasonal factors. Moderate or strong coefficient correlations also occurred for the remaining parameters; however, the least significant correlations were found for the DOC and NO_3_. 

A preliminary assessment of the results indicates that the parameters value are strongly diverse depending on the land use or seasons. Therefore, each parameter will be separately interpreted, taking into account these factors to determine the spatio-temporal variation of the Bzura River water chemistry.

#### 3.1.2. Temperature

The average annual water temperature of the Bzura River at individual sites (except for sites 4–6) was higher in 2018 (Tables S3 and S4). Based on the CA, it can be seen that the division of sites was quite similar in both years (Figure S1). In 2018, cluster I (sites 4–11 with site 4 as a hotspot) and cluster II can be distinguished as containing sub-clusters IIa (sites 3, 12, 13) and IIb (sites 1 and 2). In 2019, site 4 was separated from both clusters. Generally, cluster I was characterized by the narrower temperature ranges at a given site compared with cluster II. Higher temporal variability for cluster II was also confirmed by the higher CVs (except for site 1). Site 4 had the lowest CV values and the highest average temperature in both years. Additionally, in 2019, the highest temperature was recorded at this location in almost all months. Site 4 is below the wastewater treatment plant, so the increase in the river water temperature was due to the treated wastewater discharge [3,33]. 

The points included in cluster I (Figure S1) are located in the areas of diversified use (agricultural lands, urban and rural areas, communication routes). On the other hand, points 1–3 are located in the Łódź agglomeration, and points 12 and 13 in rural areas, away from the main communication routes. Among many factors, insolation has a great influence on the water temperature [34]. Trees and shrubs growing on the river banks reduce sunlight. In turn, urbanization may cause an increase in river water temperature because of wastewater discharge and surface runoff from the paved areas [35], hence the difference in the annual temperature variability. From November to February (2018) and from November to January (2019), when the insolation was the lowest (Table S5) [36], the temperatures for cluster II were lower than for cluster I. The influence of warmer waters discharged from numerous wastewater treatment plants located on this section of the river is probably of importance here. The influence of wastewater on water temperature was more noticeable in the winter than in the summer. Therefore, with the increasing insolation, the temperatures for both clusters were similar.

The temperature distribution in individual months was different in both years (Figure S2). In 2018, two clusters were distinguished. In the colder months (November–March, cluster I), low water temperatures (below 10 °C) were recorded (especially in January and February). In the warmer months (April–October, cluster II), the water temperature was above 10 °C and often close to 20 °C (especially from June to August). In 2019, three clusters of months were distinguished. From December to February (cluster I), the water temperature was below 10 °C, and from June to August (cluster II), the temperatures were in the range of 10 °C–20 °C. The third cluster was the remaining months (autumn and spring), when the temperature exceeded 10 °C but did not drop below 5 °C.

The correlation coefficients between the average monthly water and air temperatures (Table S5) were 0.98 and 0.96 (*p* < 0.01) in 2018 and 2019, respectively. This confirms the significant influence of the air temperature on the water temperature in the river [37].

In both years, there were visible differences in the water temperature variability (CV) depending on the month. In the months with the lowest temperatures, variability was low, moderate, or high. On the other hand, in the warmer periods, the CVs did not exceed 20% (low variability). This suggests that the influence of anthropogenic factors causing the increase in the water temperature is more pronounced in the colder months.

#### 3.1.3. Dissolved Oxygen

The average annual oxygen concentrations for most sites (except for sites 4 and 12) were higher in 2018. However, the difference did not exceed 0.8 mg/L (Tables S3 and S4). In both years, the distribution of measurement sites was similar (Figure S3). Based on CA, site 4 (hot-spot) and two clusters can be distinguished: I (sites 3, 5, 12, 13) and II (sites 1, 2, 6–11). In the entire period under study, the lowest DO concentrations were recorded in site 4 (a minimum value of 1.1 mg/L was recorded after landfill ignition), and the maximum concentrations did not exceed 10 mg/L. The reason for the low content of DO was the inflow of organic pollutants [38], the source of which was waste landfills and a wastewater treatment plant located above this site. In the case of the remaining sites, the maximum DO concentrations reached higher values (Tables S3 and S4). In cluster II, the minimal DO concentrations were higher than in cluster I. Lower temporal variability in cluster II was also confirmed by lower CVs. In the summer, a sharp decrease in oxygen concentration was observed at points from cluster I compared with the other points. In the case of points 5, 12, and 13, which are located in agricultural areas, this suggests the influence of pollutants from surface runoff from the fields and the decomposition of vegetation [39]. In addition, point 5 is burdened with contaminants arriving from point 4. Point 3 is located in the city behind a large pond in the city park. The water oxygenation level can be lowered both as a result of plant decomposition and increased insolation of the reservoir [40]. Higher concentrations of oxygen in cluster II were related to the location in the source area, where there are no significant sources of pollution (points 1 and 2), an increased flow, and the presence of thresholds (points 6–11). These thresholds cause an increase in water oxygenation, which is why quite high oxygen concentrations were observed in this section despite the presence of potential pollution emitters (e.g., wastewater treatment plants, surface runoff from urban and rural areas). 

According to the CA for the months (Figure S4), two clusters were distinguished. Cluster I covered the period of November–March (2018) and November–April (2019), with high oxygen concentrations at all points except point 4 (Tables S6 and S7). The remaining months formed cluster II, in which the oxygen concentrations were lower at all sites. In 2018, the DO contents (average and for individual sites) were clearly lower in June–September (subcluster IIa) than in April, May, and October (cluster IIb). In 2019, subcluster IIa includes months with the lowest DO concentrations and the highest air temperatures (June and August), as shown in Table S5. The inverse relationship between monthly average air temperatures and oxygen concentrations was confirmed by the correlation coefficients, which were −0.96 and −0.97 (*p* < 0.01) in 2018 and 2019, respectively. A strong relationship also occurred between the water temperatures and the oxygen concentration. The correlation coefficient for both years was −0.97 (*p* < 0.01), which proves the dominant influence of the natural factor (i.e., temperature) on the DO content in the Bzura water. Similar relationships were also observed by other authors [39,41].

#### 3.1.4. Dissolved Organic Carbon

As shown in Table S1, most of the analyzed water samples exceeded the limit values for class II river water (i.e., good). The average annual DOC values at sites 4 and 9–13 were higher in 2018, whereas in the remaining sites, it was higher in 2019 (Tables S3 and S4). The division of measurement sites according to CA was different in both years (Figure S5). A common feature was the identification of point 4 as a hotspot, where the highest DOC concentration was recorded almost every month for two years. As in the case of oxygen, this is probably due to the location of this point being below the wastewater treatment plant and landfill. In addition, two clusters of points were distinguished: cluster I (points 1–3, 7–10) and cluster II (5, 6, 11–13) in 2018, and cluster I (points 1, 2, 9–13) and cluster II (points 3, 5–8) in 2019. In both years, cluster I contained points with lower average annual DOC values compared with cluster II (Tables S3 and S4). In both years, points 1, 2, 9, and 10 were in cluster I, whereas points 5 and 6 belonged to cluster II. Points 1 and 2 are located in the upper, least polluted part of the river, and they were characterized by the lowest DOC values in both years. In the case of sites 5, 6, 9, and 10, the main sources of organic substances are urban and communal pollutants; however, points 5 and 6 are additionally burdened with a large load of pollutants arriving from point 4. Therefore, the DOC content in these points depends on the sources that undergo less variability over time. Points 7, 8, and 11–13 were classified in a different cluster each year. In these places, the Bzura flows through rural areas, where factors such as fertilization and irrigation of agricultural fields or mowing of meadows have a significant impact on the DOC level, which may change due to, for example, crop diversification [42]. A particularly clear change in the DOC levels was observed for points 11–13. These sites are in the lower course of the river, which is characterized by stronger soil erosion associated with increased water flow in the river. This process is an important source of organic carbon [43]. In addition, flooding frequently occurs in the fields, which is conducive to the leaching of organic matter from the soil. CVs for individual sites indicate low and moderate variability. However, it is not possible to indicate a clear relationship between the distribution of sites according to CA and CVs.

According to the literature, the concentration of DOC in river water is strongly related to the amount of rainfall. In Polish surface waters, the highest DOC is most often observed in the summer (intensive rainfall causes leaching of organic matter from the soil), and it is lowest in the winter (poor precipitation) [43]. However, the impact of anthropopressure, and the climate, which cause increasing differences between annual precipitation sums, contribute to significant changes in these relationships. Anthropogenic changes in the water cycle can cause an increase in the DOC, especially in urban and agricultural areas [44]. The DOC concentrations in the Bzura confirm the significant impact of factors disturbing the natural temporal pattern. Each year, a different distribution of months according to the CA was observed, which is difficult to interpret (Figure S6). It was probably caused not only by intensive surface runoff but also by the dilution of pollutants during heavy rainfall, the rapid decomposition of organic matter at higher temperatures [45], the irregular discharge of wastewater from the treatment plants, as well as the influx of other household and agricultural pollutants.

#### 3.1.5. Electrical Conductivity

The average annual values of EC in the Bzura River were higher for almost all sites (except sites 5 and 6) in 2018. In both years, the CA results separated two clusters of points (Figure S7). In 2018, these were sites 1–3 (cluster I) and 4–13 (cluster II), whereas in 2019, cluster I comprised sites 4–6 and the remainder of the sites were in cluster II. Despite these differences, five identical sub-clusters (A-E) can be distinguished in both years. These are sites 1–3 (A), 4 (B), 5 and 6 (C), 7–11 (D), and 12 and 13 (E). Points 1–3 with the lowest conductivity values are located in the upper course of the river, where there are no significant sources of pollution. In each month, the highest conductivity was observed at point 4. Strong water pollution at this point was caused by the proximity of the landfill and by the discharge of wastewater from the nearby treatment plant [46]. Points 5 and 6 had lower conductivity than point 4 but were still at a high level. These sites are burdened with pollutants from point 4, although the EC values have been reduced due to dilution. Each month there was a clear decrease in conductivity at point 5. At point 6, there was an increase in conductivity in some months, probably due to the impact of the wastewater treatment plant located above this point. At points 7–11, a slight dilution continued, and the EC values stabilized. However, especially at site 11, the conductivity periodically increased, which can be associated with the runoff of pollutants from the nearby road with heavy traffic. At sites 12 and 13, the conductivity tended to increase. The largest differences between the annual averages in 2018 and 2019 were also recorded here (Tables S3 and S4). In 2018, there was long-term flooding, which was conducive to the leaching of dissolved substances from agricultural fields. In addition, the weir above site 12 is used to dam up water in the summer to irrigate the land (arable fields, meadows, peat bogs). The CVs for most sites indicated a low temporal variability of the EC and the impact of similar sources of pollution in individual points during the year.

Average monthly EC values were higher in 2018 in the period from November to June (especially from November to March). In turn, from July to October, higher conductivity occurred in 2019 (Tables S6 and S7). This is due to the differences in the amount of precipitation. In general, the period from November to June was classified as dry and very dry in 2018, whereas in 2019, it was mainly classified as wet (only November and June were very dry) [47,48]. This indicates an increase in conductivity during dry periods (Figure S8). Using the CA in 2018, two clusters were distinguished, with cluster I covering the period from November to March and also June, when the highest conductivity was observed in this particular year, and cluster II containing the remaining months (when the conductivity was lower). In 2019, a different trend occurred, as cluster I contained the months from November to May with monthly EC averages not exceeding 700 µS/cm, whereas the values obtained from June to October (cluster II) were slightly higher. In 2018, in the months from cluster II, it was drier and warmer, but there were also heavy rains, which caused a dilution effect [49]. In turn, flooding occurred from November to March, which led to the systematic leaching of dissolved substances and increased water conductivity in the river. In 2019, the period from June to October was drier and warmer when higher EC values were recorded. From November to May, there was more rainfall, but due to low temperatures, the migration of dissolved substances was slowed down. In other Polish rivers, higher conductivity was usually observed in the winter [50,51]. In the case of the Bzura River, such a situation only occurred in 2018. This suggests that the conductivity does not strictly depend on the season, but on a set of factors related to both land use and hydrological and meteorological conditions. The latter are subject to increasing diversification under the influence of climate. CV values from 22% to 35% were recorded over two years, indicating a moderate spatial variability of conductivity for each month.

#### 3.1.6. Nitrates

The average annual concentrations of nitrates (NO_3_) were higher in 2018 at almost all sites (except point 6) (Tables S3 and S4). The largest differences occurred for points 12 and 13, which may be related to the flooding that took place in 2018. In both years, CA distinguished two clearly separated clusters: cluster I, which included sites 1–11; and cluster II, which included sites 12 and 13 (Figure S9). This division was directly related to land use; cluster II included areas that were intensively used for agriculture, where the main sources of NO_3_ are manure and nitrogen fertilizers [52]. Nitrates are not strongly adsorbed by most soils; therefore, they are leached and migrate, e.g., to surface waters [53]. The CVs were within a very wide range, and the annual variability for individual sites was determined from moderate to exceptionally high. However, there was no clear trend related to CA clustering.

The CA showed inter-annual differences between the clustering of months (Figure S10). In both years, the following clusters of months were distinguished with lower NO_3_ concentrations: November–January and March–August in 2018 and December and March–October in 2019. In these months, the NO_3_ content was usually below 20 mg/L. Despite low concentrations in these clusters, characteristic differences can be observed depending on the land use. In 2018, in the November–January subcluster, higher levels of NO_3_ occurred at sites 11–13, which was associated with the leaching of NO_3_ as a result of flooding. Lower concentrations of NO_3_ in the remaining sites (1–10) could be caused by dilution. In this part of the river, the source of NO_3_ is mainly wastewater. In March and April, increased levels of NO_3_ occurred in the section from point 4 to point 6 (effect of the wastewater treatment plant), but the highest concentrations were determined for point 12, which could be related to spring fertilization [54]. The subcluster containing May and June was a period of intensive growth of plants using NO_3_ [55], which significantly reduces their concentration in river water. In July and August, there were local increases in NO_3_ concentrations but without a clear spatial trend, which was probably disturbed by heavy rainfall (combined effect of dilution and surface runoff) (Table S5). In 2019, several characteristic subclusters were also distinguished. In March and April, as in 2018, the effect of spring fertilization in agricultural areas was observed. The subclusters containing October and May as well as June and December were the months with the lowest concentrations of NO_3_ during the year. This was due to both the intensive vegetation (May and June) and the dilution effect (May and December). On the other hand, in the period of July–September, low concentrations were generally observed, but single increases were visible, especially below the wastewater treatment plants. With the low water level at that time, the impact of wastewater became visible [56].

In both years, the clusters of months generally characterized by high concentrations of NO_3_^−^ were also distinguished. In 2018, these were February, September, and October, and in 2019, they were November (clearly different from the other months) as well as January and February. The reason for the increase in the NO_3_ contents in the winter (January, February) was low temperature, reduced biological activity [56], and the mineralization of organic matter [50]. High concentrations of NO_3_ in September and October 2018 and also in November 2019 could be the result of intense summer rainfall (Table S5) and subsequent flooding in the autumn. The concentration of NO_3_ had been growing since July 2018, but a clear increase was only visible in the autumn, when vegetation stopped. In addition, autumn organic fertilization may have contributed to the increase in the amount of NO_3_ [54,57]. 

The general trend towards high concentrations of NO_3_ in the winter and low concentrations in the summer was also observed in other Polish catchments [55,56]. In the case of the Bzura, seasonality was most visible in areas that were intensively used for agriculture, where it was observed that the beginning of the growing season marked a significant decrease in NO_3_ concentrations. The simultaneous impact of several factors may disrupt seasonality [52]. In the section from point 1 to 11, it was masked by wastewater discharges or runoff from roads and lawns, among other sources. 

#### 3.1.7. Phosphates

The differences between average annual concentrations of phosphates (PO_4_) do not exceed 0.2 mg/L (Tables S3 and S4). In both years, the CA revealed two clusters; however, they were formed by different sites (Figure S11). In 2018, points 4 and 6–9 were included in cluster I, while the remaining points formed cluster II. In cluster II, two subclusters can be distinguished: IIa (points 10–13) and IIb (1–3, 5). The highest concentrations were found in cluster I, where the PO_4_ came from wastewater treatment plants (4 and 6), runoff from roads (7 and 8), and urban pollution (9) [58,59]. Lower concentrations occurred in subcluster IIa, which mainly covered agricultural areas, where the main source of PO_4_ came from fertilizers [60]. In addition, higher concentrations were also affected by the presence of a treatment plant (10) and a road with heavy traffic (11). The lowest PO_4_ levels were recorded in subcluster IIb, which included points located upstream, and point 5, where the pollution from point 4 was probably diluted. In 2019, the lowest concentrations also occurred in the section of the river from site 1 to 3. The other sites formed a cluster characterized by higher concentrations. Unlike in 2018, points 5 and 10–13 were also included in this cluster. In 2019, higher concentrations were observed in point 4 (especially in the June–October period), which could have resulted in higher PO_4_ levels in site 5. In turn, flooding in the autumn period could have resulted in an increase in PO_4_ concentrations in points 12 and 13. However, in the summer of 2019, the water levels were lower than they had been in 2018 (Table S8) [61], which contributed to a lower dilution effect in points 10–13. Most CV values indicate moderate temporal variability in both years, and no clear association with CA clustering can be found.

The CA also showed differences in the clustering of months (Figure S12). In 2018, the cluster characterized by lower concentrations was formed for the months from November to March and July, whereas higher concentrations of PO_4_ occurred more often in the remaining months. Low PO_4_ levels in the winter (increased flows) and high levels in the summer months (lower flows) were also observed in other rivers [62,63]. In the case of Bzura, July was an exception due to the heavy rains that occurred. According to [64], the concentration of PO_4_ increases after storms, but in the Bzura, it was probably diluted due to a more than 10-fold increase in the flow. In 2019, higher concentrations occurred in November, April, May, July, and August, whereas it was lower in the remaining months. The differences compared with 2018 were mainly due to different climatic conditions (Tables S5 and S8). For example, November 2019 was characterized by exceptionally low rainfall, which led to an increase in PO_4_ concentrations in the river. On the other hand, the decrease in the PO_4_ concentration in September was the result of dilution caused by the increase in precipitation.

Despite the above-mentioned anomalies, in both years there was a statistically significant correlation between the average monthly concentrations of PO_4_ and the water temperatures (0.70 and 0.60 (*p* < 0.05) in 2018 and 2019, respectively). As in the case of clustering sites, there was no correlation between the CV coefficients and the CA results, and moderate spatial variability was observed for most months (Tables S6 and S7).

#### 3.1.8. Chlorides, Sodium, and Potassium

The annual and monthly average concentrations of chlorides, sodium, and potassium were strongly correlated. The correlation coefficients between Na and Cl in both years ranged from 0.94 to 0.99 (*p* < 0.01); between K and Cl, they varied from 0.64 (*p* < 0.05) to 0.96 (*p* < 0.01). The stronger correlation between Na and Cl suggests that Cl in river water was mainly in the form of sodium chloride [65]. 

The average annual concentrations of Na, Cl, and K were higher for almost all sites in 2019 (Tables S3 and S4). The greatest inter-annual differences occurred in the section from point 4 to point 6. The CA showed a similar division of sites for Na and K in both years (Figures S13 and S15). For K, cluster I (points 4–13) and cluster II (1–3) were distinguished. In cluster I, site 4 can be additionally distinguished, which differed from the others. For Na, there was a similar division, but cluster I consisted of sites 5–13, because site 4 (hotspot) was more clearly separated from both clusters. For Cl (Figure S17), a similar division can be observed in 2018. In 2019, however, two clusters were formed: I (points 4–6) and II, in which two subclusters IIa (1–3) and IIb (7–13) were distinguished. In the section from point 1 to point 3, as in the case of conductivity, the lowest concentrations of Na, Cl, and K occurred. This confirmed the low water pollution at these sites. All three parameters are components of water that come from both natural and anthropogenic sources [2,66]. High concentrations in the section from point 4 to point 13 (especially in point 4, where, apart from two months, the highest concentrations of these parameters occurred in both years) indicate pollution from human activity. Elevated Na concentrations are often observed in urban areas and near wastewater discharges. A similar situation is the case for Cl, which may additionally come from leachate from landfills [67] and surface runoff from agricultural fields. In the case of K, mineral fertilizers are often the main source [2].

The average monthly concentrations of Na, Cl, and K, apart from a few exceptions, were higher in 2019 (Tables S6 and S7). The greatest differences for Na and Cl were observed in January, July, and August and for K in July. The CA showed inter-annual differences for all three parameters (Figures S14, S16 and S18). However, similarities can be observed between the above parameters in a given year. In 2018, June, September, and October (additionally May for K) formed a cluster, with the highest concentrations of the above-mentioned ions. In 2019, the highest concentrations were observed from June (July for Na) to October, and additionally in January (Cl, Na) and February (Cl). The remaining months were characterized by lower concentrations. According to the literature, the highest concentrations of Na, Cl, and K occur most often in the summer months, which is associated with low precipitation and high temperatures [68,69]. Our research supports this relationship; however, in addition, we observed several factors that disrupted the seasonal pattern and caused inter-annual differences. One of them was heavy rains in 2018, which resulted in the dilution of substances dissolved in river water in July and August. Another was the differences in the amount of snowfall and snow cover formation. The snow cover only formed in January 2019, which was the month with the most days of snowfall. For this reason, this month had elevated Cl and Na concentrations recorded, unlike in 2018. This was related to the use of de-icing salts on roads, which is the main cause of river water salinity in urban catchments in northern latitudes [69]. Chloride levels also remained high in February, as the use of NaCl for road de-icing causes a long-term increase in Cl concentrations, which in some cases can also be seen in the summer [69]. Another important factor is the changes in the length of the growing season, which have been observed over the last few decades in Europe, North America, and East Asia [70]. In Poland in 2018, a significant acceleration of vegetation was observed [71]. In 2019, the growing season also started earlier, but the cooling that appeared in May slowed it down [72]. These changes affected the differences in seasonal potassium concentrations, as in 2018, elevated levels were observed from May, whereas in 2019, elevated levels were only observed from June. This was probably the result of later spring fertilization with potassium fertilizers in 2019.

#### 3.1.9. Bicarbonates, Calcium, and Magnesium

Calcium, magnesium, and bicarbonates naturally occur in water [73], but their elevated concentrations may also indicate anthropogenic sources and are often correlated with the type of land use [74]. Strong positive correlations (from 0.79 to 0.96, *p* < 0.01) between the mean annual concentrations of HCO_3_, Ca, and Mg indicated that they came from a similar source. A significant correlation (0.67, *p* < 0.05) of monthly averages only occurred in 2018 between Ca and Mg. This suggests that the above-mentioned parameters show similar spatial variability and that seasonal variability greatly varies.

Average annual Ca concentrations were higher in 2018, with the greatest difference being noted in point 13 (Tables S3 and S4). The averages of Mg and HCO_3_ were only higher in 2018 for some points. Based on the Ca concentrations, the CA showed a different distribution of sites in both years (Figure S19). A common feature was the separation of cluster I (points 12 and 13; additionally point 11 in 2018), where the highest concentrations in individual months were most often found. The remaining sites were included in cluster II, in which several subclusters can be distinguished but without a clear connection with land development. Moreover, in 2018, the temporal variability for both clusters can be described as moderate; in 2019, it can be described as low or moderate for cluster II and moderate for cluster I. There were also inter-annual differences for Mg (Figure S21). In 2019, three clusters were distinguished: I (points 12 and 13), II (3-11), and III (1 and 2). In 2018, point 13 was separated as a hotspot, and the remaining points were clustered together. It was possible to distinguish a subcluster (points 1 and 2) that clearly differed from the others. A common feature in both years was the separation of points with the lowest (points 1 and 2) and highest (point 13) concentrations of Mg. In 2018, temporal variability can mostly be described as moderate, whereas in 2019, it was moderate in points 11-13 and low or moderate at other sites. In the case of HCO_3_, inter-annual differences were also observed (Figure S23). In both years, two clusters were separated, but with different sites. In 2018, cluster I included points 4, 12, and 13, and in 2019, it additionally included points 5 and 6. Cluster I was characterized by higher concentrations than cluster II. In both years, the lowest concentrations were in point 1 and the highest in 4, 12, and 13. Temporal variability can be mainly described as low (moderate was only at point 1 in both years and at point 3 in 2018). Mg and Ca can come from wastewater and fertilizers, which is why their elevated concentrations are observed in urban and agricultural areas [66,69]. The concentrations of Ca and Mg in surface waters in agricultural areas depend to a large extent on the drainage system used because drainage ditches cause less Mg and Ca outflow from the soil than sewage pipes [74]. Calcium is also leached from building materials [29]. In addition, in recent years, an increase in Mg concentrations has been observed due to the use of de-icing agents [29]. In our research, particularly high concentrations of Ca and Mg occurred in agricultural areas near sites 12 and 13, which are located in heavily drained floodplains. The origin of Ca and Mg from fertilizers at these points was also confirmed by correlations with the annual concentrations of NO_3_. In 2018, there was a strong correlation for both Ca-NO_3_ (0.86, *p* < 0.01) as well as Mg-NO_3_ (0.79, *p* < 0.01); in 2019, the correlation coefficients were lower and amounted to 0.74 (*p* < 0.01) and 0.68 (*p* < 0.05), respectively. Higher levels of Ca and Mg were also observed below wastewater treatment plants, in urban areas, and near roads. In the case of HCO_3_, elevated concentrations also occurred in agricultural areas, which was caused by the use of fertilizers that disturb the natural acid–base balance [73]. High levels of HCO_3_ were also reported below point 4, where the water was highly conductive and low in oxygen. These are the factors that indirectly cause the increase in HCO_3_ in water because under such conditions an excess of CO_2_ is formed, which causes the dissolution of carbonates [1]. An increase in the concentration of HCO_3_ in water can also be caused by the weathering of building materials [73].

In 2018, in the period from November to January and in September and October, the monthly averages were higher for all three parameters, whereas in 2019, they were higher in March and July (Tables S6 and S7). The CA showed differences in the clustering of months (Figures S20, S22 and S24). It is difficult to determine a general seasonal pattern because the measured concentrations did not show a clear relationship with the meteorological and hydrogeological conditions. Increased concentrations of these analytes were observed in the summer [51]. In turn, according to [75], the concentrations of Ca and Mg in river water were mainly the highest in the winter months. In general, many factors influence Ca, Mg, and HCO_3_ concentrations. Flow rate is an important factor as dilution occurs during the periods of increased flow [76,77]. In the case of the Bzura, it was most visible for Ca and HCO_3_ in July 2018. A similar effect was not observed for the remaining periods of increased flow. The level of Ca and Mg in river water is also affected by vegetation, as during the growing season, their increased accumulation in plants is noted [74]. Such a relationship only occurred in 2018 in the section from points 11 to 13, where from November to February, particularly high concentrations of Ca were observed in Bzura compared with the summer months. However, increased levels of Ca and Mg may also result from the use of de-icing agents [69], which was observed in January 2019. For HCO_3_, the effect of temperature was noticeable. In both years, elevated concentrations of HCO_3_ were mainly noted in the summer months. This is related to the release of larger amounts of CO_2_ with the increase in temperature [76]. The increase in bicarbonate concentrations in water may also result from weathering, which is taking place more and more intensively due to climate change [73]. In 2018, the highest concentrations of HCO_3_ occurred from November to March in points 12 and 13, whereas in 2019 it occurred from March to June and in August and September in points 4–6 and 13. For Ca and Mg, there was also a significant change in the spatio-temporal variability of these parameters. This may have been due to the lowered pH of the water in 2019 [26], as acidification may cause a change in the seasonal pattern [77].

#### 3.1.10. Spatial Variability in 2019

**Figure 2:**
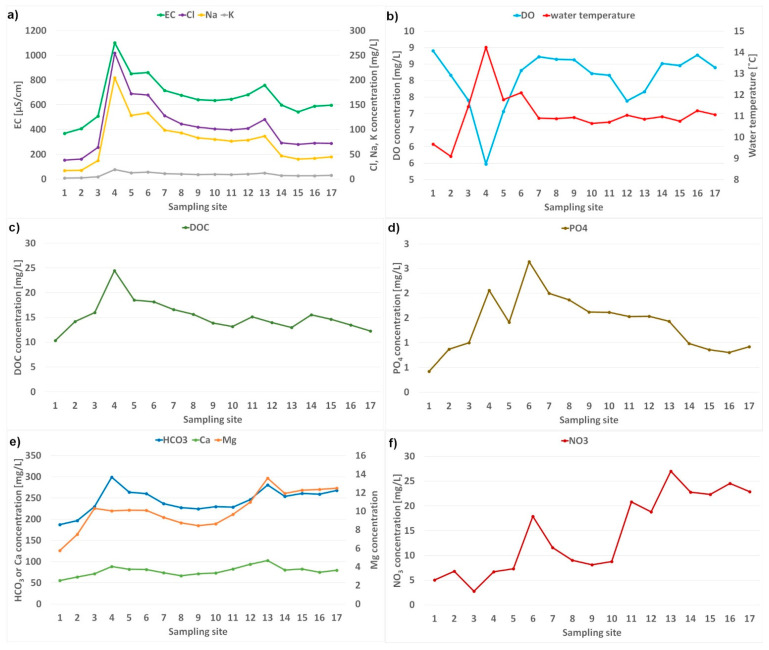
Spatial variability of the determined parameters in 2019. Annual mean values of (**a**) electrical conductivity, sodium, chlorides, and potassium; (**b**) dissolved oxygen and water temperature; (**c**) dissolved oxygen carbon; (**d**) phosphates (**e**) bicarbonates, calcium, magnesium, and (**f**) nitrates.

Based on the annual mean values of the determined parameters in 2019 for 17 sampling sites, the spatial variability on the 120 km section of the Bzura River was assessed (Figure 2). The lowest concentration of all parameters occurred in the upper section (sites 1–3) of the Bzura River. Site 4 (hotspot) was characterized by the highest concentrations (except for NO_3_). Additionally, the impact of wastewater treatment plants for phosphates and nitrates was noted. The concentration of most parameters decreased along the river flow (with local increases) or remained at a similar level. It indicates the lack of a significant sources of pollutants and a dilution effect at sites 14–17. The opposite trend was only observed for NO_3_; their concentration strongly increased in the lower course of the Bzura River, which is mainly linked to vegetation and agriculture. 

### 3.2. PCA and CA

#### 3.2.1. Spatial Variability

**Figure 3:**
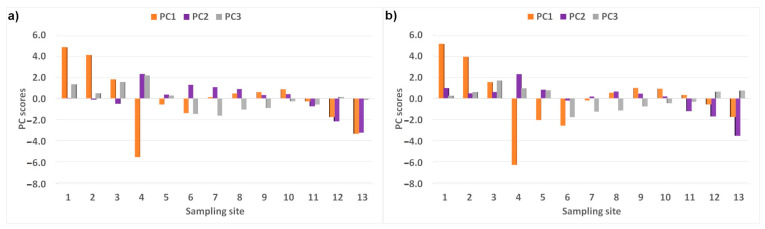
PC scores for the individual sampling points in (**a**) 2018 and (**b**) 2019.

For datasets with annual mean values, the PCA extracted three principal components (PCs) in both years (Table 4). They had eigenvalues greater than or equal to one and explained together more than 90% of the total variance. The PCs were created by similar variables in both years. PC1 was positively correlated only with DO (moderately in 2018 and strongly in 2019) and negatively strongly or moderately with the remaining variables (except for NO_3_ in 2019). PC1 may be attributed to overall river water pollution by contaminants (organic and inorganic) [78] from mixed sources. High negative PC1 scores (lower than −1 [79]), indicating strong water pollution, occurred at sites 4, 6, 12, and 13 in 2018 and 4–6 and 13 in 2019 (Figure 3). These sites are impacted by wastewater treatment plants, waste landfills, and agriculture. Sites 1–3 were characterized by low PC1 impact (PC1 scores higher than 1). PC2 had strong negative factor loadings for NO_3_ and moderate negative ones for Ca and Mg; it indicates that PC2 is related to the agricultural activity, which was proved by the PC2 scores, which were lower than −1 at sites located in the intensively farmed areas (sites 12 and 13 in 2018 and 11–13 in 2019). PC3 was created by DO and PO_4_ (with negative strong or moderate loadings) and additionally by the positively moderately correlated temperature in 2018. This component is probably mainly influenced by the wastewater treatment plant [59]. PC3 scores were the lowest (indicating high DO and PO_4_ concentrations) at sites 6–8 in both years. It is associated with the wastewater treatment plant (causes a higher PO_4_ level) as well as the thresholds (the cause of the increase in water oxygenation) above site 6. Additionally, the temperature correlation with PC3 in 2018 and the highest PC3 scores at site 4 suggest the influence of wastewater discharge on water temperature [33]. 

**Figure 4:**
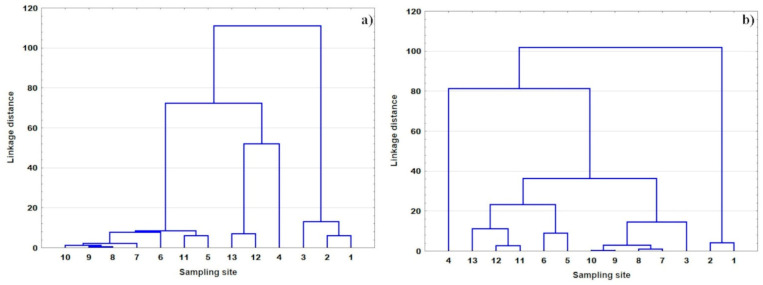
Dendrograms showing sampling sites clustering in (**a**) 2018 and (**b**) 2019.

Following the CA (Figure 4), the cluster including sites 1 and 2 (also site 3 in 2018), characterized by the lowest pollution, and site 4 (hotspot) were distinguished in both years. These sampling sites were also extracted for the majority of parameters (EC, temperature, DO, DOC, PO_4_, Cl, Na, K, HCO_3_) by the CA, which was separately performed for each parameter. In 2019, the remaining points were in one cluster, whereas in 2018, they formed two clusters. This indicates a greater distinction in 2019 between the sites located under the strong agricultural impact and the Bzura River section from sites 5 to 11, where mixed pollutants sources existed. This clustering, in opposition to the CA that was separately performed for the individual parameters, did not allow for the identification of the sources of pollution in detail.

#### 3.2.2. Temporal Variability

By following the datasets with monthly mean values, five principal components (PCs) can be extracted in both 2018 and 2019 (Table S9). Together, they explained more than 90% of the total variance. The eigenvalues of PC5 and PC4 (in 2018) were lower than one; however, each of them explains more than 5% of the total variance [80], therefore they have also been considered. 

**Figure 5:**
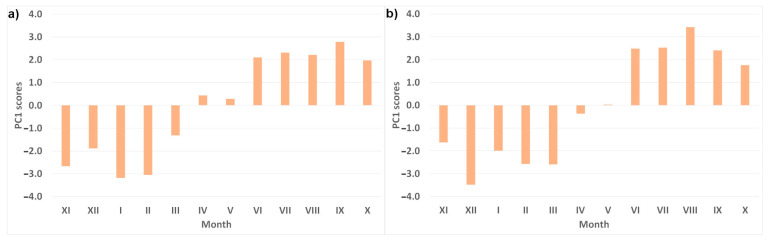
PC1 scores for the individual months in (**a**) 2018 and (**b**) 2019.

In both years, PC1 was strongly or moderately linked to EC (negatively in 2018 and positively in 2019), temperature, PO_4_, Cl, Na, and K (positively) and to DO and Mg (negatively). NO_3_ was additionally moderately negatively correlated in 2018, and HCO_3_ was moderately positively correlated in 2019. Strong factor loadings with the temperature and PC1 scores lower than –1 from November to March and higher than 1 from June to October (Figure 5) indicate that this component is mainly associated with temperature changes throughout the year. The remaining PCs were created by different variables in both years and are not easily interpreted (Figure S25). For example, in 2018, PC2 is probably associated with dilution (as a result of heavy rains) or increased concentrations (as a result of flooding). Contrary to 2018, PC2 in 2019 can represent the use of de-icing agents (especially in January), when the snow cover was formed.

**Figure 6:**
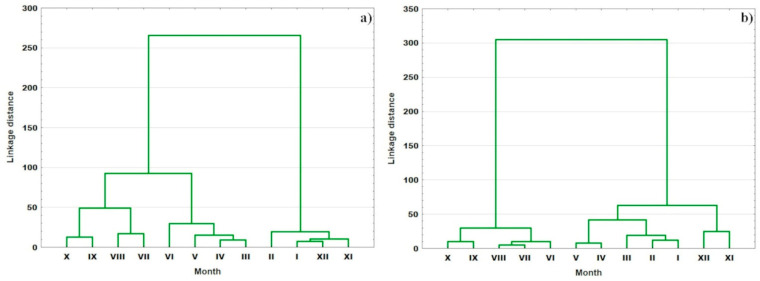
Dendrograms showing the months clustering in (**a**) 2018 and (**b**) 2019.

Following the CA (Figure 6), two clusters were distinguished in both years; however, they were created by different months. In 2018, the first cluster contained months from November to February, and the remaining ones created the second cluster. In 2019, it was from November to May and from June to October, respectively. This clustering is mainly associated with the water temperature. This CA proved that the most important factor influencing the seasonal variability was temperature. However, (similar to the sampling sites clustering based on the annual mean values) it also masks other factors, which are also non-negligible.

### 3.3. Water Quality Index

WQIs were calculated for the threshold values of the class II water shown in Table A2. For the comprehensive evaluation of the Bzura River water quality, the above-described parameters, the trace metals (Zn, Cu, Pb, Ni, and Cd) concentrations, and the total dissolved solids (TDS) [26] were used in the calculations. The summary of WQIs for individual sites in 2018 and 2019 is shown in Table 5.

In terms of the mean WQIs (Table 5), several sections of the river can be distinguished.

**Section I**—sites 1 and 15–17 (good water quality [27]). Site 1 located in the source zone of the Bzura River in the Wzniesienia Łódzkie Landscape Park was characterized by good water quality. Considering the WQI values in the following months of 2018 and 2019 (Table S10), this point is the least contaminated, despite the fact that it is situated in the city of Łódź, by the street with relatively heavy traffic. The protective function is played by deciduous trees, which constitute a barrier limiting the impact of emissions from urban areas and from transportation. Water quality deteriorated in the period of July–September in 2018, whereas in the following year, it was only poor in August. As the highest water and air temperatures were recorded in these months, it can be assumed that the reason for the deterioration of the water quality was the concentration of pollutants as a result of evaporation.

Considering the threshold values for the water of class II [31], it was found that at site 1 throughout the period, the water quality for most parameters met the requirements of class I or II. In the case of chlorides, their concentration slightly exceeded the limit value for class II in 2019 and was a maximum of 42.7 mg/L (annual mean 38.2 mg/L). In both years, the permissible P-PO_4_ concentration was exceeded. In this case, a high content of phosphorous can be explained by the intensive decomposition of organic matter in the park, soil erosion, and, to a lesser extent, atmospheric precipitation [81,82]. The average annual WQIs at sites 15–17 located in the lower course of the river also corresponded to good quality water, but greater variability of some parameters during the year was observed. In terms of temperature, oxygen content, TDS, and heavy metals, water can be classified as class I or II. The values of DOC, EC, Cl, and P-PO_4_ exceeded the limits for class II in most samples. In the case of other parameters, the threshold values were exceeded in the winter and spring, and the high content of HCO_3_ persisted even until July–August. The seasonal variability of these parameters did not clearly depend on the land use, as sites 15 and 16 are located in the city, whereas point 17 is in a rural area.

**Section IIa**—sites 2, 3, and 14 (in 2019) had poor water quality (WQI = 103–119) [27]. Point 2 is located on the border of Łódź and Zgierz, which is part of the Łódź agglomeration. This site is located by the national road 71 (with heavy traffic), which connects the agglomeration with the A1 and A2 motorway junctions. Site 3 is in the center of Zgierz on the border of industrial and low-rise residential areas with a local heating system. The average WQIs of these points slightly exceeded the range of values for good quality water (Table 5), but the WQIs in individual months indicated a deterioration of the water quality. This is especially noticeable in the summer. In 2019, poor water conditions were also recorded in September and October, of which the result may be due to a lower dilution of water pollutants, associated with less rainfall during this period. The WQIs in the summer and autumn of 2019 were significantly higher than in the corresponding months of 2018 (Table S10). The poor water quality (>class II) at sites 2 and 3 was most affected by the values of DOC, Cl (sites 2 and 3 in 2019 and site 3 in 2018), Ca (in 2018), and P-PO_4_. Site 14 is located in agricultural areas (arable fields, meadows). Considering the WQI values in individual months, slightly different variability of this parameter can be noticed. Poor water quality occurred in the winter months, in late spring, and in August 2019 and resulted from exceeding the limits of DOC, Cl, P-PO_4_, HCO_3_; in the winter and spring, it was also due to exceeding the limits of N-NO_3_, Ca and Mg.

**Section IIb**—sites 5 and 9–13 (poor water quality WQI = 141–191) [27]. Water pollution in this section was higher than in section IIa. The river flows here through areas of diversified use (small towns, such as Ozorków, Łęczyca, and Kutno with local and household sewage treatment plants, as well as rural areas, arable fields, and floodplains), and site 5 is located below site 4 (hot-spot) in the city of Zgierz. The limits for water class II [31] of DOC, EC, Cl, and P-PO_4_ values were exceeded at all sites. In addition, at sites 5, 12, and 13, in the period of June–September, a significant reduction in the concentration of DO was observed, which was simultaneously associated with the increase in water temperature and intensive oxygen uptake by aquatic plants. In the case of the remaining parameters, only the water temperature was not exceeded at any station. Compared with the points included in Section 2a, in group IIb, a greater number of analysis results exceeded the limit values for TDS, Ca, Mg, N-NO_3_, and HCO_3_. This primarily applies to sites 12 and 13, which are located in agricultural areas.

**Section III**—sites 4 and 6–8 (very poor water quality) [27]. This section of the river receives wastewater from two large treatment plants, which are discharged above sites 4 and 6. An additional source of water pollution at site 4 is runoff from industrial waste landfills. The low assessment of water quality was mainly due to exceeding the threshold values of DOC, EC, Cl, and P-PO_4_ for class II (Table 5) at all sites. In addition, most of the results exceed the limits for TDS, Ca (sites 4, 6–8), hardness, HCO_3_ (sites 4,6), and N-NO_3_ (sites 6–8) (Table S10). In the points located below sites 4 and 6, the WQI values decreased, which should be associated with the dilution effect.

## 4. Conclusions

Our investigation, supported by statistical analysis, allowed for the determination of the spatio-temporal variability of the river water chemistry and identification of the pollution sources of the Bzura River. The results of the two-year analyses have shown a large variability of physicochemical and chemical parameters in areas subject to strong anthropopressure.

Most of the parameters retained similar spatial variability in both years. The water quality was the best in the source area (points 1–3), despite the location of these sites being in urbanized areas. Then, points 4–11 were the most exposed to pollution from mixed sources (urbanization, agriculture, transport, surface runoff, discharge of wastewater from treatment plants of different efficiency, and uncontrolled discharge of domestic sewage). Sites 4 and 6, located directly below the wastewater treatment plants, stood out in this group. Points 12 and 13 were located in intensively farmed areas, which caused, e.g., an increase in NO_3_ concentrations. Sites 14–17 in the Bzura River downstream were characterized by a decrease in the content of most of the controlled parameters, despite being located in agricultural (sites 14 and 17) or urban areas (sites 15 and 16).

All studied parameters were influenced by both climatic and anthropogenic factors:

- The river water temperature was strongly impacted by the air temperature, isolation, and urbanization; the influence of anthropopressure was more pronounced in the colder months;
- The DO concentration was mainly dependent on the temperature and plant vegetation; however, surface runoff from the agricultural and urban areas, waste landfills ignition, and discharge of wastewater were also recorded;
- The DOC content was related to the organic compounds of natural (erosion) and anthropogenic nature (wastewater treatment plants, landfills, fertilizers, and pesticides); extreme weather events disturbed the natural temporal pattern;
- The concentrations of Na, K, Cl, PO_4_, and EC were mainly associated with point sources of pollutants (wastewater treatment plants, waste landfills, roads), but the temperature, rainfalls, flooding and drought, or river flow were also very important;
- The most important factor for the NO_3_ concentration was the vegetation, which was most visible in intensively farmed areas; in the more transformed section, the impact of wastewater discharges and runoff from roads disrupted the seasonality;
- The HCO_3_, Mg, and Ca concentrations mainly increased in the agricultural areas; however, erosion (Ca, Mg), wastewater treatment plants, and runoff from roads were also important factors. These parameters did not show a clear relationship with the weather conditions.

A significant influence of climatic conditions on the level of river pollution was found. Temporal variability was determined by more factors than spatial variability. The PCA revealed that the temperature explained less than 50% of the seasonal variability. Moreover, the phenomena related to climate conditions significantly hinder the predictability of the seasonal variability of most parameters. The fluctuation in pollution level mainly results from the dilution during the periods of heavy rainfall and the concentration of pollutants during droughts. The influence of the plant vegetation period on the chemical composition of the water was also observed. This caused, for example, an increase in the content of nutrients in the winter months and a decrease during the period of increased plant development.

Multivariate statistical analyses based on datasets containing various parameters are useful in determining general environmental relationships, but they may also mask some important factors. The CA carried out separately for individual parameters showed a different variability of parameters (which are dependent on climatic conditions) in natural and transformed sections of the river. The transformed fragments were characterized by fluctuation, which was more difficult to forecast.

As mentioned in Section 3.1, throughout the research period, the values of most parameters exceeded the thresholds for class II water purity. A particularly serious threat to the Bzura River is water salinity that is mainly related to a high concentration of chloride salts, DOC, and PO_4_. The data of the state environmental monitoring from 2021 for four control points located on this section of the river testify to the persistently bad chemical conditions of the Bzura River. With the exception of temperature (class 1—very good water), the other parameters mainly correspond to class > 2 (conductivity, TDS, Cl, Ca, and N-NO_3_ on four sites; DO, DOC, P-PO_4_, total hardness, and Mg on two sites). Therefore, the water quality in the entire section of the river was defined as below good.

An important element of water resources management is a reliable assessment of the water quality. In order to carry out this task, the number of control points under the SEM should be increased. In particular, sites 4 (hotspot) and 6 require careful monitoring. However, the first SEM measurement control point is located about 13 km and 6 km below these sites, respectively. Therefore, it would be advisable to conduct a continuous monitoring of the basic parameters, e.g., EC, DO, and temperature, due to the variability of the water chemistry in these places. Additionally, it would be necessary to conduct monitoring (at least once a month) at sites 12 (located in an agricultural area and downstream of a weir, which may cause fluctuations in the water chemistry) and 13 (apart from the impact of agriculture, it is also probably burdened with pollutants delivered by one of its tributaries-the Ochnia River). In addition to an efficient monitoring system, the deficit of surface water resources requires authorities to introduce a number of systemic changes. Creating incentives for users to retain rainwater or the more widespread use of gray water, which is currently discharged into the sewage system, are examples of simple and not so expensive methods to improve the situation in Poland. An important aspect is also regulating the use of water resources in agriculture and limiting the emission of agricultural pollutants. The next step is to consistently enforce penalties for the illegal discharge of wastewater into the environment. This is particularly important in the case of enterprises whose activities may have a significant impact on the environment, as exemplified by the aforementioned ecological disaster on the Oder river.

This research proved that in the face of climate changes and the related progressive water deficit, it is necessary to pursue a rational policy in the management of water resources. Sustainable water management also includes water protection system, an integral part of which ensures efficient environmental monitoring. The water quality control methods currently used in Poland and other European countries do not meet the requirements of the early warning system, as evidenced by the recent ecological disaster on the second largest Polish river, the Oder River. It is therefore necessary to implement more effective methods of monitoring and have them be conducted with a greater participation from scientific and research institutions than before.

## Figures and Tables

**Table 1:** Statistics of the parameters obtained for the Bzura River water samples.

		2018 ^1^			2019 ^1^			2019 ^2^		
Parameter	Unit	Min	Max	Mean	Min	Max	Mean	Min	Max	Mean
EC	µS/cm	266	1297	737	327	1405	681	315	688	583
temp	°C	0.4	22.9	11.9	1.4	22.3	11.1	1.5	23.4	11.0
DO	mg/L	1.1	13.8	8.3	2.8	10.9	7.9	4.8	11.4	8.5
DOC	mg/L	6.7	43.7	16.4	7.8	35.4	15.6	9.1	23.0	14.0
NO_3_	mg/L	n.d. ^3^	112	18.1	n.d. ^3^	100	11.7	2.0	127	23.1
PO_4_	mg/L	0.02	4.1	1.5	0.2	4.1	1.5	0.2	2.1	0.9
HCO_3_	mg/L	105	430	237	128	342	239	207	296	260
Cl	mg/L	29.3	258	88.0	34.5	340	116	25.1	99.2	72.1
Na	mg/L	8.9	194	63.9	14.0	290	87.6	20.7	78.6	43.5
K	mg/L	1.4	56.9	9.1	1.5	25.5	9.8	4.1	11.2	7.1
Ca	mg/L	15.2	226	102	40.9	185	77.4	12.7	131	79.1
Mg	mg/L	2.3	34.6	10.0	4.5	19.1	9.5	7.5	20.1	12.3

^1^ Results for 13 sampling points. ^2^ Results for 4 additional sampling points. ^3^ not detected.

**Table 2:** Inter-annual Pearson and Spearman correlation coefficients at individual sampling sites.

Site	EC	temp	DO	DOC	NO_3_	PO_4_	HCO_3_	Cl	Na	K	Ca	Mg
1	−0.74 **	0.85 ^a,^**	0.58 *	−0.34 ^a^	0.50 ^a^	−0.10 ^a^	0.06 ^a^	0.52	0.22	0.63 *	−0.05	0.00
2	−0.46	0.82 **	0.51	0.04 ^a^	0.06 ^a^	0.27	0.29	0.32 ^a^	0.30 ^a^	0.70 *	−0.27 ^a^	0.13
3	0.07 ^a^	0.84 ^a,^**	0.91 **	−0.16	−0.18 ^a^	0.61 *	0.31 ^a^	0.11 ^a^	0.15 ^a^	0.15	−0.09	0.01
4	−0.56	0.88 ^a,^**	0.90 **	−0.19	0.28 ^a^	0.52 ^a^	0.16 ^a^	−0.02	0.02	0.36 ^a^	−0.01	−0.08
5	−0.36	0.95 ^a,^**	0.88 **	0.34	0.04 ^a^	0.14	0.32 ^a^	−0.04 ^a^	−0.09 ^a^	0.48 ^a^	−0.08	−0.21
6	0.19	0.92 **	0.85 **	−0.26	0.06 ^a^	−0.17	0.23	0.25 ^a^	0.17 ^a^	0.56 ^a^	−0.05	−0.21 ^a^
7	−0.60 *	0.90 **	0.81 **	−0.65 *	−0.15	0.25	0.07 ^a^	−0.40	−0.16	0.39 ^a^	−0.25	−0.60 ^a,^*
8	−0.70 *	0.88 **	0.89 **	−0.14 ^a^	0.18	0.23	0.31	−0.05	0.02	0.31 ^a^	−0.25	−0.28 ^a^
9	−0.64 *	0.89 **	0.83 **	0.23 ^a^	0.14 ^a^	0.36	0.14	0.27 ^a^	0.25	0.25 ^a^	−0.13	0.07 ^a^
10	−0.52	0.91 **	0.81 **	0.33	0.23	0.56	0.35 ^a^	0.26 ^a^	0.08 ^a^	0.49	0.18	−0.22 ^a^
11	−0.75 ^a,^*	0.86 **	0.83 **	−0.40 ^a^	0.69 ^a,^*	0.43	−0.13 ^a^	0.15 ^a^	0.20	0.74 ^a,^**	0.20	0.45 ^a^
12	−0.23	0.88 **	0.90 **	0.03	0.60 ^a,^*	−0.11 ^a^	0.17 ^a^	0.42 ^a^	0.51 ^a^	0.56 ^a^	0.50 ^a^	0.08
13	−0.53	0.85 **	0.86 **	−0.31 ^a^	0.45 ^a^	−0.24	−0.53	0.63 *	0.71 ^a,^*	0.85 **	0.20	−0.14

^a^ Spearman coefficients, strong correlation ≥ 0.7 [32] are bolded. * Correlations are significant at *p* < 0.05. ** Correlations are significant at *p* < 0.01.

**Table 3:** Inter-annual Pearson and Spearman correlation coefficients for the individual months.

Month	EC	Temp	DO	DOC	NO_3_	PO_4_	HCO_3_	Cl	Na	K	Ca	Mg
XI	0.88 **	0.29 ^a^	0.59 ^a,^*	−0.10 ^a^	0.42 ^a^	0.80 **	0.87 **	0.95 ^a,^**	0.77 ^a,^**	0.91 **	0.66 ^a,^*	0.79 **
XII	0.83 **	0.70 ^a,^**	0.49 ^a^	0.61 *	0.54 ^a^	0.55 ^a^	0.79 **	0.85 **	0.82 **	0.89 **	0.63 ^a,^*	0.78 ^a,^**
I	0.56 *	0.81 **	0.09 ^a^	0.49	0.92 ^a,^**	0.80 ^a,^**	0.83 **	0.64 *	0.75 **	0.98 **	0.86 ^a,^**	0.53
II	0.98 **	0.97 **	0.44 ^a^	0.54	0.35 ^a^	0.66 *	0.83 **	0.94 ^a,^**	0.97 **	0.90 ^a,^**	0.69 ^a,^**	0.93 ^a,^**
III	0.97 **	−0.18 ^a^	0.36 ^a^	0.49 ^a^	0.76 ^a,^**	0.39	0.44	0.94 ^a,^**	0.93 ^a,^**	0.98 **	0.88 **	0.78 ^a,^**
IV	0.98 **	−0.13	0.34 ^a^	0.68 ^a,^*	0.68 ^a,^*	0.76 **	0.76 **	0.98 **	0.95 ^a,^**	0.97 **	0.56 *	0.92 **
V	0.99 **	0.64 ^a,^*	0.90 ^a,^**	0.26 ^a^	0.82 ^a,^**	0.63 *	0.74 **	0.95 ^a,^**	0.95 ^a,^**	0.99 **	0.85 **	0.85 ^a,^**
VI	0.99 **	0.80 **	0.86 **	0.78 ^a,^**	0.27 ^a^	0.81 **	0.97 **	0.99 **	0.99 ^a,^**	0.99 **	0.69 **	0.85 ^a,^**
VII	0.87 **	0.57 *	0.72 **	0.42 ^a^	0.02 ^a^	0.22	0.29	0.86 **	0.90 **	0.95 **	0.59 **	0.92 **
VIII	0.82 **	0.15	0.84 **	0.35 ^a^	−0.36	0.86 **	0.70 **	0.85 **	0.85**	0.75 ^a,^**	0.51 ^a^	0.63 *
IX	0.97 **	−0.14	0.66 *	0.66 *	0.26	0.04	0.50	0.97 **	0.99 **	0.98 **	0.21 ^a^	0.93 **
X	0.98 **	0.84 **	0.64 ^a,^*	0.10	0.34 ^a^	0.62 *	0.38	0.99 **	0.98 **	0.98 **	0.48	0.84 ^a,^**

^a^ Spearman coefficients, strong correlation ≥ 0.7 [32] are bolded. * Correlations are significant at *p* < 0.05. ** Correlations are significant at *p* < 0.01.

**Table 4:** Factor loadings of variables calculated based on the annual average values of the determined parameters for 2018 and 2019.

	2018			2019		
	PC1	PC2	PC3	PC1	PC2	PC3
Eigenvalue	7.7	2.1	1.4	8.3	2.1	1.0
% of the total variance	64.0	17.6	11.4	69.6	17.4	8.7
EC	**−0.98**	0.11	−0.08	**−0.99**	0.08	−0.10
temp	**−0.75**	0.00	*0.50*	**−0.91**	0.31	0.10
DO	*0.59*	−0.14	**−0.75**	**0.73**	−0.19	−*0.64*
DOC	**−0.95**	0.07	0.15	**−0.83**	0.47	0.07
NO_3_	*−0.50*	**−0.77**	−0.16	−0.31	**−0.89**	−0.21
PO_4_	*−0.54*	0.45	−*0.65*	**−0.75**	−0.01	−*0.62*
HCO_3_	**−0.93**	−0.22	−0.02	**−0.96**	−0.16	0.14
Cl	**−0.90**	0.41	−0.03	**−0.96**	0.23	−0.09
Na	**−0.87**	0.47	−0.06	**−0.95**	0.22	−0.16
K	**−0.91**	0.27	−0.20	**−0.97**	0.02	−0.20
Ca	**−0.74**	*−0.63*	−0.20	**−0.74**	−*0.62*	0.22
Mg	**−0.76**	*−0.61*	−0.03	−*0.66*	−*0.65*	0.23

Strong correlation are bolded. Moderate correlation are italic.

**Table 5:** The WQIs calculated based on the annual average parameters.

Site No.	2018	2019
1	80	61
2	103	106
3	118	119
4	200	219
5	141	162
6	253	280
7	230	219
8	202	204
9	191	179
10	174	172
11	165	160
12	168	157
13	151	146
14	no data	104
15	no data	89
16	no data	89
17	no data	93

Water quality: good—green; poor—yellow; very poor—orange.

## Data Availability

All data are presented in the article.

## Supplementary Materials

The following supporting information can be downloaded at https://www.mdpi.com/article/10.3390/ijerph20043032/s1: Table S1. Sampling site characteristics; Table S2. Percentage of results that exceeded the threshold values for class II [31]; Table S3. Results of the determined parameters in the Bzura River water for the individual sampling sites in 2018; Table S4. Results of the determined parameters in the Bzura River water for the individual sampling sites in 2019; Table S5. Weather conditions (monthly mean values of the air temperature, sunshine duration, and precipitation for the study area [36]); Table S6. Results of the determined parameters in the Bzura River water for the individual months in 2018; Table S7. Results of the determined parameters in the Bzura River water for the individual months in 2019 (for 13 sampling sites); Table S8. Hydrological conditions (parameters read on the sampling days based on the measurement data on the gauging station at point 11 [61]); Table S9. Factor loadings of variables calculated based on the monthly average values of the determined parameters for 2018 and 2019; Table S10. WQI values and water quality assessment [27] for individual months and measurement points in 2018 and 2019; Figure S1. Dendrograms depicting the clustering of sampling sites in (a) 2018 and (b) 2019 for temperature; Figure S2. Dendrograms depicting the clustering of months in (a) 2018 and (b) 2019 for temperature; Figure S3. Dendrograms depicting the clustering of sampling sites in (a) 2018 and (b) 2019 for dissolved oxygen; Figure S4. Dendrograms depicting the clustering of months in (a) 2018 and (b) 2019 for dissolved oxygen; Figure S5. Dendrograms depicting the clustering of sampling sites in (a) 2018 and (b) 2019 for DOC; Figure S6. Dendrograms depicting the clustering of months in (a) 2018 and (b) 2019 for DOC; Figure S7. Dendrograms depicting the clustering of sampling sites in (a) 2018 and (b) 2019 for electrical conductivity; Figure S8. Dendrograms depicting the clustering of months in (a) 2018 and (b) 2019 for electrical conductivity; Figure S9. Dendrograms depicting the clustering of sampling sites in (a) 2018 and (b) 2019 for nitrates; Figure S10. Dendrograms depicting the clustering of months in (a) 2018 and (b) 2019 for nitrates; Figure S11. Dendrograms depicting the clustering of sampling sites in (a) 2018 and (b) 2019 for phosphates; Figure S12. Dendrograms depicting the clustering of months in (a) 2018 and (b) 2019 for phosphates; Figure S13. Dendrograms depicting the clustering of sampling sites in (a) 2018 and (b) 2019 for sodium; Figure S14. Dendrograms depicting the clustering of months in (a) 2018 and (b) 2019 for sodium; Figure S15. Dendrograms depicting the clustering of sampling sites in (a) 2018 and (b) 2019 for potassium; Figure S16. Dendrograms depicting the clustering of months in (a) 2018 and (b) 2019 for potassium; Figure S17. Dendrograms depicting the clustering of sampling sites in (a) 2018 and (b) 2019 for chlorides; Figure S18. Dendrograms depicting the clustering of months in (a) 2018 and (b) 2019 for chlorides; Figure S19. Dendrograms depicting the clustering of sampling sites in (a) 2018 and (b) 2019 for calcium; Figure S20. Dendrograms depicting the clustering of months in (a) 2018 and (b) 2019 for calcium; Figure S21. Dendrograms depicting the clustering of sampling sites in (a) 2018 and (b) 2019 for magnesium; Figure S22. Dendrograms depicting the clustering of months in (a) 2018 and (b) 2019 for magnesium; Figure S23. Dendrograms depicting the clustering of sampling sites in (a) 2018 and (b) 2019 for bicarbonates; Figure S24. Dendrograms depicting the clustering of months in (a) 2018 and (b) 2019 for bicarbonates; Figure S25. Score values of PC2, PC3, PC4, and PC5 in (a,b,c,d) 2018 and (e,f,g,h) 2019 for the individual months.

## Appendix A

**Table A1:** Detailed results of the RM analysis (n = 6).

	Metal Concentrations [mg/L]			
Sub-Sample	Ca	Mg	Na	K
1	93.2	25.9	44.0	3.2
2	94.7	25.7	46.3	2.9
3	93.5	25.6	44.0	3.4
4	95.3	26.0	44.1	2.5
5	94.3	26.0	43.9	3.1
6	94.3	26.1	45.0	2.9
Obtained values [mg/L]	94.2 ± 0.8	25.9 ± 0.2	44.6 ± 1.0	3.00 ± 0.33
Certified values [mg/L]	95.4 ± 7.3	25.4 ± 2.0	43.0 ± 4.2	3.51 ± 0.29
Recovery [%]	98.8	102	104	85.5

## Appendix B

**Table A2:** The threshold values of the physicochemical and chemical parameters [31].

Parameter	Unit	Permissible Value	
		Class I	Class II
Temperature	°C	≤22	≤24
Dissolved oxygen (DO)	mg O_2_/L	≥7.0	≥6.6
Dissolved organic carbon (DOC)	mg C/L	≤9	≤10.8
Electrical conductivity (EC) at 25 °C	µS/cm	≤411	≤553
Total dissolved solids (TDS)	mg/L	≤282	≤375
Chlorides	mg/L	≤14	≤34.5
Calcium	mg/L	≤72.0	≤81.7
Magnesium	mg/L	≤12.1	≤12.8
Total hardness	mg CaCO_3_/L	≤225	≤266
Alkalinity	mg CaCO_3_/L	≤185	≤205
N–NO_3_	mg/L	≤1.6	≤2.5
P–PO_4_	mg/L	≤0.065	≤0.101
Zinc	mg/L	≤1	≤1
Copper	mg/L	≤0.05	≤0.05
Cadmium	µg/L	≤0.45	≤0.45
Lead	µg/L	≤14	≤14
Nickel	µg/L	≤34	≤34

Total hardness was calculated based on the sum of the Mg and Ca concentrations; alkalinity was calculated based on the HCO_3_ concentrations.
