# Supplementary material for: Combined Effect of Climate and Anthropopressure on River Water Quality
Source: Int J Environ Res Public Health. 2023 Feb 9;20(4):3032. doi: 10.3390/ijerph20043032 (PMC9960277; doi:10.3390/ijerph20043032)
Supplement: Supplementary file 1 [file ijerph-20-03032-s001.zip › ijerph-2135055-supplementary.pdf]

Table S1. Sampling site characteristics.

Sampling Site	Characteristics
1	Source area of the river; protection area (the Łódzkie Hills Landscape Park)
2	Residential area; near the national road 71
3	Urban area (the city of Zgierz)
4	Below the wastewater treatment plant (for the city of Zgierz), waste landfill, and industrial park; suburban area of the city of Zgierz
5	Agricultural land
6	Below the wastewater treatment plant (for the city of Aleksandrów Łódzki); meadows and agricultural land
7	Near the motorway A2; meadows and agricultural land
8	Near the local, busy road; meadows, and agricultural land
9	Urban area (the city of Ozorków)
10	Below the wastewater treatment plant (for the city of Ozorków); agricultural land
11	Near the provincial road; meadows, and wasteland, below the city of Łęczyca
12	Agricultural land (cultivation of vegetables and cereals); floodplains
13	Agricultural land (cultivation of vegetables and cereals); floodplains
14	Agricultural land (cultivation of cereals)
15	Near the national road 92; warehouse and service areas
16	Urban area (the city of Łowicz)
17	Agricultural land (orchard crops)

Table S2. Percentage of results that exceeded the threshold values for class II [31].

	2018	2019	2018 and 2019
**EC**	22	35	28
**DO**	21	21	21
**DOC**	92	84	88
**NO_3**	50	22	37
**PO_4**	97	94	96
**HCO_3**	35	35	35
**Cl**	87	100	94
**Ca**	79	33	56
**Mg**	21	11	16

Table S3. Results of the determined parameters in the Bzura River water for the individual sampling sites in 2018.

	Parameter	**1**	**2**	**3**	**4**	**5**	**6**	**7**	**8**	**9**	**10**	**11**	**12**	**13**
**EC** [μS/cm]	min	266	297	398	790	616	640	582	544	530	465	423	566	689
	max	509	557	762	1297	962	1110	870	876	874	828	912	1088	1140
	mean	**408**	**445**	**554**	**1115**	**826**	**843**	**774**	**739**	**707**	**689**	**728**	**839**	**917**
	CV [%]	17.3	17.3	20.3	14.7	12.6	14.5	11.0	12.7	13.4	16.0	20.9	21.5	16.7
**temp** [˚C]	min	1.6	0.4	1.8	5.8	3.1	3.5	2.9	2.9	1.7	1.7	1.4	0.8	0.4
	max	19.8	19.3	21.0	20.6	19.3	19.2	18.6	18.6	19.3	19.5	21.4	22.2	22.9
	mean	**11.9**	**10.7**	**12.2**	**13.8**	**11.5**	**11.9**	**11.4**	**11.6**	**11.7**	**11.6**	**11.9**	**12.3**	**12.6**
	CV [%]	62.2	69.6	67.9	45.9	56.4	52.8	53.0	51.9	55.5	56.6	60.1	65.5	65.2
**DO** [mg/L]	min	6.6	5.7	2.1	1.1	4.7	6.4	7.1	7.0	6.4	5.1	5.1	1.6	2.4
	max	13.8	12.6	13.1	9.3	11.6	12.4	12.8	11.7	12.5	12.1	12.5	12.1	13.0
	mean	**8.9**	**8.6**	**7.6**	**5.1**	**7.7**	**8.8**	**9.3**	**9.3**	**9.3**	**8.5**	**9.0**	**7.3**	**8.4**
	CV [%]	25.4	27.1	50.7	51.2	29.8	21.3	19.2	16.7	20.4	27.8	27.5	43.1	37.8
**DOC** [mg/L]	min	6.7	10.0	8.7	17.5	12.8	14.1	10.9	12.1	10.5	10.3	11.5	7.2	10.5
	max	14.6	16.8	19.5	31.0	19.6	20.8	20.7	20.1	33.5	24.1	37.4	20.7	43.7
	mean	**10.3**	**13.2**	**14.6**	**25.0**	**16.5**	**17.6**	**14.9**	**15.3**	**16.0**	**15.0**	**17.8**	**16.4**	**20.7**
	CV [%]	26.9	17.1	22.1	17.1	14.0	13.5	18.0	17.2	40.1	25.2	40.1	24.7	41.9
**NO_3** [mg/L]	min	0.9	n.d.	n.d.	1.3	0.2	3.9	5.7	3.7	1.7	3.2	4.2	7.3	7.5
	max	37.3	23.2	13.8	26.5	17.5	32.5	24.2	16.4	63.6	35.7	61.4	95.1	112
	mean	**9.5**	**6.9**	**5.4**	**13.3**	**9.2**	**14.2**	**11.9**	**10.8**	**17.2**	**15.5**	**26.9**	**45.4**	**45.0**
	CV [%]	136	133	78.4	63.9	61.0	69.3	49.5	39.8	113	73.1	71.6	73.4	77.9
**PO_4** [mg/L]	min	0.2	0.5	0.02	1.2	0.6	1.1	1.1	1.0	0.8	1.0	0.7	0.8	1.0
	max	3.0	1.4	1.9	2.9	2.4	4.1	3.4	2.8	2.4	2.3	2.6	4.0	1.7
	mean	**0.7**	**1.9**	**1.0**	**1.8**	**1.2**	**2.4**	**2.1**	**1.8**	**1.7**	**1.6**	**1.5**	**1.5**	**1.3**
	CV [%]	111	31.8	56.3	34.6	39.4	39.4	36.0	33.5	31.5	25.3	35.3	54.3	17.5
**HCO_3** [mg/L]	min	106	162	145	211	184	185	197	199	190	140	105	200	239
	max	253	267	430	312	284	290	251	243	263	245	271	317	334
	mean	**150**	**199**	**236**	**287**	**250**	**254**	**233**	**224**	**224**	**221**	**233**	**269**	**297**
	CV [%]	28.4	15.6	30.8	9.2	9.5	12.7	7.3	6.1	8.2	13.2	18.0	13.5	10.5
**Cl** [mg/L]	min	30.1	29.3	37.9	105	78.0	70.9	64.7	71.7	67.4	57.5	48.2	67.4	68.2
	max	37.2	35.5	70.0	258	201	184	131	127	134	116	104	118	149
	mean	**32.8**	**32.5**	**46.8**	**181**	**111**	**116**	**99.4**	**94.4**	**88.3**	**80.8**	**75.0**	**86.1**	**98.7**
	CV [%]	6.5	5.6	17.9	53.5	33.2	34.4	20.7	20.1	25.0	24.1	23.0	20.2	27.1
**Na** [mg/L]	min	13.7	14.0	22.1	84.4	57.1	53.5	48.6	52.6	48.1	43.7	8.9	33.9	26.2
	max	17.7	18.7	44.7	195	152	151	106	103	98.2	90.2	88.8	98.0	121
	mean	**15.6**	**15.9**	**27.0**	**145**	**81.4**	**91.1**	**78.2**	**72.9**	**65.6**	**61.1**	**50.3**	**60.1**	**66.5**
	CV [%]	8.1	9.7	22.6	21.8	34.8	39.5	23.4	23.4	26.3	26.9	44.0	37.4	49.8
**K** [mg/L]	min	1.4	2.1	3.6	14.2	7.4	8.2	8.5	7.9	7.1	6.7	6.5	6.5	6.2
	max	2.1	3.8	4.9	21.9	17.2	19.2	15.0	13.4	11.9	12.0	57.0	12.8	17.8
	mean	**1.8**	**2.9**	**4.4**	**17.2**	**10.1**	**12.1**	**10.5**	**9.7**	**8.7**	**8.8**	**12.5**	**9.0**	**10.8**
	CV [%]	11.4	20.0	9.1	17.8	29.3	34.2	20.4	20.0	19.9	20.7	112.7	22.8	35.1
**Ca** [mg/L]	min	27.0	36.9	31.7	38.0	32.3	37.3	44.4	43.6	35.6	26.7	15.2	28.0	55.7
	max	120	139	137	157	184	178	174	160	169	156	157	196	226
	mean	**74.0**	**80.6**	**94.9**	**104**	**107**	**99.7**	**101**	**98.6**	**102**	**94.4**	**110**	**127**	**144**
	CV [%]	35.1	34.4	29.5	27.9	32.6	35.2	30.4	29.0	33.8	34.2	34.0	35.8	32.2
**Mg** [mg/L]	min	2.3	3.0	4.4	4.7	5.1	5.1	4.6	4.4	4.2	4.5	4.9	6.6	7.1
	max	9.0	12.5	17.6	18.5	17.4	15.9	14.7	16.0	13.2	16.2	21.6	25.9	34.6
	mean	**5.5**	**6.7**	**10.0**	**11.0**	**10.8**	**10.3**	**8.7**	**8.7**	**8.4**	**8.8**	**10.5**	**13.0**	**17.4**
	CV [%]	35.8	42.2	36.4	35.0	34.9	30.7	35.6	34.3	31.0	37.9	44.3	46.5	46.7

Table S4. Results of the determined parameters in the Bzura River water for the individual sampling sites in 2019.

	Parameter	**1**	**2**	**3**	**4**	**5**	**6**	**7**	**8**	**9**	**10**	**11**	**12**	**13**	**14**	**15**	**16**	**17**
**EC** [μS/cm]	min	327	352	435	737	573	540	473	451	444	440	420	507	549	479	315	451	486
	max	424	438	731	1405	1048	1137	914	867	817	781	749	932	1035	688	664	682	674
	mean	**369**	**407**	**509**	**1101**	**851**	**862**	**716**	**677**	**642**	**637**	**645**	**682**	**759**	**599**	**544**	**590**	**598**
	CV [%]	8.5	5.7	14.8	16.6	16.2	19.9	15.2	16.0	16.0	14.5	13.2	15.0	15.2	12.2	19.5	13.4	10.4
**temp** [°C]	min	2.6	1.4	2.3	6.5	4.9	4.5	4.0	4.0	3.6	3.2	2.8	2.0	1.6	1.5	1.5	1.8	1.5
	max	16.8	18.8	21.3	21.7	19.9	20.8	19.8	20.2	19.5	19.4	19.4	22.1	22.3	22.8	22.3	23.4	22.5
	mean	**9.7**	**9.1**	**11.4**	**14.2**	**11.8**	**12.1**	**10.9**	**10.9**	**10.9**	**10.7**	**10.7**	**11.0**	**10.9**	**11.0**	**10.8**	**11.3**	**11.1**
	CV [%]	47.2	58.4	59.9	38.0	43.8	46.7	46.9	48.7	49.4	51.0	54.4	60.9	62.9	59.8	60.1	59.1	59.5
**DO** [mg/L]	min	7.1	5.7	3.0	2.8	3.9	6.2	6.3	6.4	6.1	5.6	5.7	3.2	3.6	4.8	5.8	5.8	6.0
	max	10.1	9.8	10.5	8.0	8.9	10.1	10.8	10.8	10.8	10.5	10.9	10.6	10.2	11.4	10.4	10.7	10.3
	mean	**8.9**	**8.2**	**7.4**	**5.5**	**7.1**	**8.3**	**8.7**	**8.6**	**8.6**	**8.2**	**8.2**	**7.4**	**7.7**	**8.5**	**8.5**	**8.8**	**8.4**
	CV [%]	11.2	13.9	34.7	32.4	22.2	15.5	16.3	16.2	16.6	18.0	19.4	29.7	25.8	25.8	16.8	17.9	16.5
**DOC** [mg/L]	min	7.8	9.1	10.6	12.7	12.1	12.6	10.6	10.6	10.3	9.9	9.0	10.3	10.3	11.9	9.8	9.9	9.1
	max	16.5	24.5	27.5	35.4	28.5	25.6	24.7	27.7	24.3	17.9	28.5	21.6	15.3	18.8	23.0	16.2	15.7
	mean	**10.3**	**14.2**	**16.0**	**24.4**	**18.5**	**18.1**	**16.6**	**15.6**	**13.9**	**13.2**	**15.1**	**14.0**	**13.0**	**15.5**	**14.6**	**13.5**	**12.3**
	CV [%]	24.6	27.5	29.7	25.3	23.8	23.2	24.1	31.2	29.1	19.7	35.4	23.4	12.6	12.9	23.1	13.8	15.7
**NO_3** [mg/L]	min	1.5	n.d.	n.d.	n.d.	1.1	1.7	3.4	2.4	1.7	1.0	2.2	1.9	n.d.	4.4	1.6	3.4	4.1
	max	12.7	57.5	14.2	26.7	22.7	62.1	18.5	18.6	20.7	14.5	101	56.1	66.2	104	113	126	106
	mean	**5.0**	**6.8**	**2.8**	**6.7**	**7.3**	**17.9**	**11.6**	**9.0**	**8.1**	**8.8**	**20.8**	**18.8**	**27.0**	**22.8**	**22.3**	**24.5**	**22.9**
	CV [%]	68.4	248	150	107	76.0	83.6	44.3	62.0	65.4	48.2	129	99.6	99.0	134	148	148	129
**PO_4** [mg/L]	min	0.2	0.2	0.2	1.2	0.8	0.8	1.0	0.9	0.8	0.8	0.7	0.7	0.2	0.4	0.3	0.2	0.3
	max	0.7	1.4	2.4	3.2	2.3	4.1	3.1	3.1	2.2	2.2	2.1	3.2	2.4	1.9	1.3	1.6	2.1
	mean	**0.4**	**0.9**	**1.0**	**2.1**	**1.4**	**2.6**	**2.0**	**1.9**	**1.6**	**1.6**	**1.5**	**1.5**	**1.4**	**1.0**	**0.9**	**0.8**	**0.8**
	CV [%]	38.8	48.6	56.4	33.6	37.3	43.9	35.4	39.0	27.2	25.9	28.9	46.2	41.9	46.1	39.1	50.1	56.2
**HCO_3** [mg/L]	min	128	175	192	247	227	220	198	191	196	194	183	210	227	207	214	215	238
	max	321	240	343	341	298	310	279	259	251	253	252	265	330	277	296	278	287
	mean	**187**	**197**	**230**	**299**	**264**	**260**	**237**	**227**	**225**	**229**	**229**	**246**	**281**	**254**	**261**	**259**	**273**
	CV [%]	33.4	8.7	16.9	10.4	8.8	11.0	9.2	9.0	7.7	8.0	8.8	7.7	10.2	8.1	9.2	8.0	6.0
**Cl** [mg/L]	min	34.5	36.5	50.6	182	116	95.8	86.8	63.2	74.9	71.7	81.0	81.0	90.4	25.1	53.1	55.6	54.1
	max	42.7	55.1	169	340	248	283	193	172	146	135	128	178	193	99.2	92.8	95.4	93.7
	mean	**38.2**	**40.6**	**64.4**	**255**	**173**	**170**	**128**	**112**	**105**	**101**	**99**	**103**	**120**	**73.4**	**70.1**	**72.7**	**72.2**
	CV [%]	6.9	12.9	51.6	19.8	24.8	32.3	23.2	26.0	20.2	18.4	15.9	25.2	24.2	26.2	16.9	15.1	14.2
**Na** [mg/L]	min	14.0	14.7	23.2	127	81.0	73.3	58.2	51.9	48.4	49.0	50.2	50.0	40.8	25.2	20.7	21.9	23.4
	max	20.5	33.0	128	290	185	221	145	154	130	119	131	187	197	77.5	72.8	73.4	78.6
	mean	**17.4**	**17.9**	**37.2**	**205**	**129**	**134**	**98.8**	**93.4**	**83.4**	**80.3**	**77.0**	**79.0**	**87.0**	**47.2**	**40.2**	**41.8**	**44.7**

	CV [%]	11.7	28.2	77.4	25.0	29.1	37.1	28.3	30.6	28.8	28.2	34.5	48.1	55.5	28.7	36.0	33.6	30.9
	min	1.5	1.9	3.6	14.1	8.8	8.3	7.1	6.4	6.1	6.7	6.3	7.3	6.1	5.0	4.1	4.4	5.2
**K**	max	2.2	3.3	6.3	25.5	17.5	20.8	15.2	14.9	13.3	14.0	13.0	17.6	20.5	9.7	9.4	9.4	11.2
[mg/L]	mean	**1.9**	**2.6**	**4.6**	**19.4**	**12.6**	**14.2**	**11.0**	**10.2**	**9.4**	**9.7**	**9.3**	**9.9**	**12.1**	**7.2**	**6.6**	**6.7**	**7.8**
	CV [%]	13.8	18.5	19.2	19.9	23.8	32.5	22.6	24.9	22.1	26.3	28.7	36.8	18.5	18.5	22.3	20.2	20.3
	min	40.9	49.8	48.6	68.6	62.1	60.8	54.5	47.8	47.1	50.6	52.8	55.7	58.6	48.5	53.1	12.7	44.2
**Ca**	max	66.6	85.0	92.5	108	106	107	93.5	82.9	89.8	107	143	178	185	131	120	125	125
[mg/L]	mean	**55.6**	**63.5**	**71.4**	**88.5**	**81.8**	**81.2**	**73.6**	**66.7**	**71.2**	**73.2**	**82.5**	**93.8**	**103**	**79.8**	**82.4**	**74.6**	**79.5**
	CV [%]	13.7	16.5	20.8	17.8	18.7	20.4	19.0	18.3	18.5	21.4	28.2	35.6	34.2	29.8	26.3	40.7	29.2
	min	4.5	5.5	8.0	8.3	8.6	8.7	7.5	6.5	7.3	6.7	6.8	6.6	8.3	7.9	7.6	8.7	7.5
**Mg**	max	8.1	10.8	13.0	12.0	12.2	13.3	13.4	13.2	12.5	13.6	16.2	17.9	19.1	17.9	18.8	19.9	20.1
[mg/L]	mean	**5.8**	**7.5**	**10.3**	**10.0**	**10.1**	**10.1**	**9.3**	**8.8**	**8.5**	**8.6**	**9.7**	**11.0**	**13.6**	**11.9**	**12.3**	**12.3**	**12.5**
	CV [%]	18.6	20.1	15.2	11.6	10.6	14.4	16.2	19.1	17.7	21.9	30.7	35.4	30.6	23.9	25.9	27.9	27.0

Table S5. Weather conditions (monthly mean values of the air temperature, sunshine duration, and precipitation for the study area [36]).

	Temperature [˚C]		Sunshine duration [h]		Precipitation total [mm]	
	**2018**	**2019**	**2018**	**2019**	**2018**	**2019**
**XI**	5	3.5	35	60	40	< 10
**XII**	2	1	30	20	35	50
**I**	1	-2	35	20	27	40
**II**	-4	1	75	105	<10	30
**III**	0	5.5	125	125	15	30
**IV**	12.5	9.5	260	260	35	<20
**V**	17	11.5	320	180	50	50
**VI**	18	21.5	250	370	30	20
**VII**	19.5	17.5	240	220	85	35
**VIII**	19.5	20	290	260	40	35
**IX**	14.5	13.5	220	160	45	65
**X**	9.5	10	160	130	60	<30

Table S6. Results of the determined parameters in the Bzura River water for the individual months in 2018.

	Parameter	XI	XII	I	II	III	IV	V	VI	VII	VIII	IX	X
**EC** [μS/cm]	min	509	475	493	411	460	380	383	418	365	344	369	266
	max	1168	1282	1129	1297	1292	1086	1164	1271	915	790	1106	980
	mean	**822**	**868**	**818**	**811**	**803**	**707**	**710**	**813**	**599**	**568**	**716**	**612**
	CV [%]	23.7	25.4	22.6	29.4	27.9	25.9	26.9	30.0	28.5	23.8	29.6	33.3
**temp** [°C]	min	5.2	1.9	0.4	0.4	3.8	12.4	16.2	18.1	17.5	17.5	15.4	9.6
	max	9.4	6.5	5.8	5.8	8.6	17.1	21.6	22.9	21.1	21.0	19.2	17.5
	mean	**6.8**	**4.9**	**3.1**	**2.2**	**5.7**	**14.4**	**18.3**	**19.9**	**19.4**	**18.9**	**16.7**	**12.8**
	CV [%]	17.0	34.6	43.8	66.5	25.0	10.2	11.0	7.5	5.6	5.4	7.4	17.8
**DO** [mg/L]	min	7.9	7.5	9.3	8.1	6.7	3.5	3.7	1.1	1.6	2.1	3.0	4.0
	max	10.1	11.4	13.8	13.0	10.9	10.5	9.4	7.5	7.7	7.9	8.7	9.4
	mean	**9.3**	**10.1**	**11.9**	**11.7**	**10.0**	**8.4**	**7.2**	**5.5**	**5.5**	**5.7**	**6.4**	**8.0**
	CV [%]	8.6	9.5	9.3	10.8	11.5	20.3	17.9	33.9	33.8	35.8	29.5	18.2
**DOC** [mg/L]	min	13.1	12.3	13.4	7.9	12.1	7.6	11.7	6.7	7.2	9.0	10.3	7.4
	max	27.0	24.7	25.1	19.9	27.7	31.0	43.7	27.0	18.8	24.8	17.5	33.5
	mean	**16.5**	**19.0**	**19.1**	**14.6**	**17.9**	**15.2**	**19.6**	**13.9**	**13.6**	**16.1**	**13.0**	**18.3**
	CV [%]	27.6	16.0	14.4	23.6	22.7	37.9	53.0	33.3	25.4	30.4	15.0	42.4
**NO_3** [mg/L]	min	n.d.	0.3	n.d.	7.7	0.2	1.8	n.d.	0.2	3.8	2.3	9.5	3.6
	max	86.0	79.8	78.5	112	95.1	42.5	28.7	26.5	36.0	37.4	40.7	50.4
	mean	**27.1**	**17.5**	**17.9**	**38.7**	**17.1**	**16.1**	**7.8**	**5.9**	**13.1**	**14.9**	**24.6**	**15.6**
	CV [%]	112	156	140	86.7	143	63.2	95.9	115	72.7	68.9	48.5	86.9
**PO_4** [mg/L]	min	0.5	0.2	0.6	0.3	0.5	0.4	0.02	0.4	0.5	0.8	0.8	0.3
	max	1.6	2.2	3.0	1.6	2.2	3.0	4.1	2.7	2.6	2.5	4.0	3.3
	mean	**1.2**	**1.2**	**1.4**	**1.1**	**1.1**	**1.8**	**1.7**	**1.7**	**1.3**	**1.8**	**2.2**	**1.6**
	CV [%]	25.7	53.3	48.1	31.2	41.1	50.0	63.1	37.1	49.5	30.9	45.3	48.5
**HCO_3** [mg/L]	min	163	172	223	151	134	164	125	131	105	118	128	118
	max	330	328	334	314	311	299	305	308	239	290	430	312
	mean	**252**	**239**	**260**	**246**	**236**	**242**	**237**	**239**	**175**	**222**	**251**	**242**
	CV [%]	17.0	19.9	13.2	19.3	19.3	13.7	18.2	21.1	23.1	19.6	27.6	22.4
**Cl** [mg/L]	min	34.6	31.0	30.1	31.0	30.1	31.0	29.3	32.0	31.3	33.1	32.7	35.5
	max	164	166	105	189	186	180	177	218	167	128	239	258
	mean	**76.6**	**86.1**	**65.3**	**78.2**	**83.5**	**82.0**	**76.2**	**109**	**80.5**	**76.0**	**119**	**124**
	CV [%]	42.2	40.9	30.9	51.2	48.0	47.2	49.3	47.6	48.5	38.3	49.5	51.9
**Na** [mg/L]	min	14.8	15.5	15.5	13.9	15.7	13.7	14.0	14.1	14.7	8.9	15.2	16.8
	max	124	127	84.4	141	170	157	151	186	131	112	164	195
	mean	**46.3**	**60.3**	**45.2**	**53.3**	**65.5**	**57.5**	**56.5**	**85.8**	**59.6**	**53.3**	**57.6**	**95.8**
	CV [%]	59.4	50.9	39.7	65.6	60.3	62.5	62.5	56.8	57.0	58.0	50.9	56.0
**K** [mg/L]	min	1.8	1.7	1.7	2.0	1.9	1.5	1.4	1.7	1.8	1.9	2.1	1.9
	max	14.2	15.2	15.4	14.8	19.4	15.0	19.8	21.3	14.4	57.0	20.2	21.9
	mean	**7.1**	**7.9**	**7.5**	**7.3**	**8.6**	**7.2**	**9.0**	**10.8**	**8.0**	**11.9**	**12.0**	**12.1**
	CV [%]	40.9	41.3	44.5	44.5	51.2	45.4	51.9	52.3	47.5	118	47.8	50.7
**Ca** [mg/L]	min	58.7	67.9	68.3	110	78.3	45.5	80.9	120	15.2	68.7	52.5	51.8
	max	171	180	226	196	132	124	141	194	55.7	169	116	116
	mean	**103**	**101**	**114**	**134**	**104**	**99**	**106**	**161**	**34.8**	**96.4**	**88.0**	**92.8**
	CV [%]	33.7	30.5	37.9	18.6	14.7	26.1	14.9	12.7	28.7	25.3	16.2	19.6
**Mg** [mg/L]	min	8.2	3.9	5.4	4.1	4.2	2.3	7.2	4.5	4.9	4.8	5.5	6.6
	max	25.9	17.0	26.3	17.5	14.6	7.1	20.4	13.9	9.8	9.7	12.0	34.6
	mean	**16.6**	**9.2**	**14.2**	**10.1**	**8.1**	**4.7**	**13.5**	**9.0**	**6.6**	**7.1**	**8.8**	**12.0**
	CV [%]	30.9	40.6	40.2	34.1	31.8	26.9	24.8	29.2	20.5	22.0	19.4	58.5

Table S7. Results of the determined parameters in the Bzura River water for the individual months in 2019 (for 13 sampling sites).

	Parameter	XI	XII	I	II	III	IV	V	VI	VII	VIII	IX	X
**EC** [μS/cm]	min	334	327	345	338	367	315	350	389	378	410	408	399
	max	864	737	1109	975	1068	1047	1098	1206	1226	1280	1405	1198
	mean	**568**	**483**	**666**	**658**	**652**	**638**	**656**	**724**	**721**	**722**	**731**	**680**
	CV [%]	23.5	19.1	28.8	21.3	23.7	28.1	26.4	28.8	29.9	34.8	36.0	30.0
**temp** [°C]	min	8.8	1.5	1.4	1.9	5.9	5.5	10.4	14.7	12.2	16.8	11.0	12.4
	max	11.6	7.9	6.5	7.3	11.7	13.2	15.1	23.4	21.7	21.6	16.5	18.3
	mean	**9.7**	**4.4**	**2.9**	**4.1**	**7.6**	**9.2**	**11.7**	**19.2**	**17.2**	**19.6**	**12.9**	**14.4**
	CV [%]	6.9	41.2	49.8	34.9	17.4	16.8	10.3	15.7	12.9	5.3	10.7	10.3
**DO** [mg/L]	min	6.6	7.4	8.0	7.3	6.7	5.5	4.8	3.0	3.9	2.9	2.8	5.6
	max	9.5	11.3	10.9	9.9	9.8	9.9	9.3	7.2	9.1	9.2	10.6	11.4
	mean	**8.6**	**9.9**	**10.0**	**9.3**	**8.9**	**8.7**	**7.4**	**5.7**	**7.0**	**5.7**	**7.7**	**7.7**
	CV [%]	9.0	9.1	8.1	6.4	8.7	12.8	13.2	18.7	22.7	28.9	22.9	17.0
**DOC** [mg/L]	min	8.1	7.8	8.5	9.8	11.1	12.9	9.1	8.5	10.0	9.0	10.6	15.3
	max	28.5	17.9	21.3	24.0	25.0	26.8	19.7	23.0	28.3	27.2	32.0	35.4
	mean	**12.8**	**12.6**	**16.5**	**14.8**	**15.3**	**16.6**	**11.8**	**14.2**	**16.3**	**14.9**	**17.9**	**23.8**
	CV [%]	38.4	19.0	24.3	24.4	24.0	22.0	22.8	29.3	28.5	40.6	31.5	22.9
**NO_3** [mg/L]	min	12.7	0.4	2.9	1.6	1.6	0.2	0.4	0.8	2.4	1.4	0.5	0.4
	max	100.7	19.9	61.0	126.5	64.2	31.6	9.1	16.8	14.2	13.3	22.8	18.9
	mean	**33.2**	**9.2**	**22.4**	**39.5**	**23.2**	**8.4**	**3.5**	**6.8**	**7.7**	**5.7**	**7.3**	**5.9**
	CV [%]	73.3	70.8	81.7	111.7	102.8	93.7	80.7	55.9	43.0	62.5	85.2	80.4
**PO_4** [mg/L]	min	0.5	0.6	0.2	0.4	0.2	0.2	0.5	0.2	0.2	0.6	0.3	0.5
	max	3.2	3.9	3.7	1.5	1.3	4.1	2.4	3.2	3.9	3.1	2.2	2.5
	mean	**1.7**	**1.5**	**1.3**	**0.9**	**0.6**	**1.5**	**1.6**	**1.5**	**1.6**	**1.9**	**1.2**	**1.4**
	CV [%]	46.0	49.2	63.4	40.4	56.0	71.2	37.2	45.3	64.7	42.2	48.5	37.8
**HCO_3** [mg/L]	min	150	175	182	192	155	152	128	156	175	132	145	180
	max	294	287	284	296	343	326	325	333	330	329	341	293
	mean	**240**	**228**	**239**	**238**	**251**	**254**	**253**	**259**	**245**	**245**	**242**	**240**
	CV [%]	13.3	16.3	12.9	12.2	16.3	15.8	16.6	16.8	19.0	18.3	17.7	10.2
**Cl** [mg/L]	min	34.5	25.1	36.2	37.9	36.2	36.7	36.5	37.6	38.6	40.8	36.9	40.0
	max	187	182	339	220	236	236	234	267	270	286	340	258
	mean	**87.8**	**79.1**	**128**	**100**	**91.0**	**94.1**	**98.0**	**120**	**119**	**121**	**125**	**107**
	CV [%]	46.3	44.3	69.0	45.2	51.1	49.8	48.7	49.4	50.6	60.1	63.9	52.3
**Na** [mg/L]	min	14.4	15.8	17.6	17.4	14.0	15.	18.4	15.2	17.8	14.7	15.1	18.5
	max	142	127	249	149	165	198	18	224	290	239	235	251
	mean	**61.6**	**52.5**	**88.9**	**56.0**	**55.3**	**69.4**	**69.8**	**77.5**	**115**	**93.3**	**95.6**	**90.3**
	CV [%]	55.8	49.2	80.1	65.1	66.0	64.1	55.5	63.6	60.9	72.1	68.4	68.9
**K** [mg/L]	min	1.6	2.0	1.9	2.1	1.6	1.7	1.5	1.7	2.1	2.1	2.1	2.2
	max	15.4	14.1	14.8	16.1	17.0	19.0	20.3	24.1	22.3	21.6	26.5	22.1
	mean	**8.6**	**7.3**	**7.2**	**7.0**	**7.2**	**8.4**	**8.9**	**11.1**	**11.6**	**10.9**	**11.4**	**10.0**
	CV [%]	40.9	37.6	41.8	44.4	46.5	48.4	48.9	52.9	47.9	52.1	59.8	53.2
**Ca** [mg/L]	min	63.8	52.3	12.7	47.8	66.6	40.9	61.0	50.1	50.6	44.2	48.5	54.2
	max	111.4	94.1	139	131	185	107	122	81.5	97.5	75.1	107	95.4
	mean	**87.5**	**71.9**	**90.1**	**79.8**	**106**	**69.3**	**86.4**	**63.7**	**72.0**	**61.2**	**78.1**	**67.6**
	CV [%]	16.7	17.5	31.4	38.8	31.5	28.9	16.4	14.4	18.0	14.0	22.8	15.7
**Mg** [mg/L]	min	4.7	5.7	8.1	7.0	6.5	6.4	5.3	4.5	4.7	6.0	5.2	5.2
	max	10.9	11.4	17.8	20.1	19.0	16.3	18.9	11.4	12.7	12.0	11.2	11.1
	mean	**8.3**	**8.6**	**13.3**	**13.5**	**12.2**	**11.4**	**10.0**	**8.6**	**9.0**	**9.5**	**8.9**	**8.5**
	CV [%]	17.1	16.8	16.3	32.8	29.1	25.5	32.4	21.0	25.8	21.2	18.5	15.3

Table S8. Hydrological conditions (parameters read on the sampling days based on the measurement data on the gauging station at point 11 [61]).

	Water level [cm]		Flow rate [m^3/s]	
	**2018**	**2019**	**2018**	**2019**
**XI**	130	87	3.6	0.8
**XII**	136	112	4.1	2.4
**I**	113	113	2.1	2.6
**II**	115	120	2.3	3.3
**III**	100	107	1.5	2.4
**IV**	96	90	1.3	1.0
**V**	88	83	0.8	0.8
**VI**	76	71	0.6	0.5
**VII**	155	83	6.7	0.8
**VIII**	79	75	0.6	0.6
**IX**	76	81	0.5	0.7
**X**	77	91	0.6	1.1

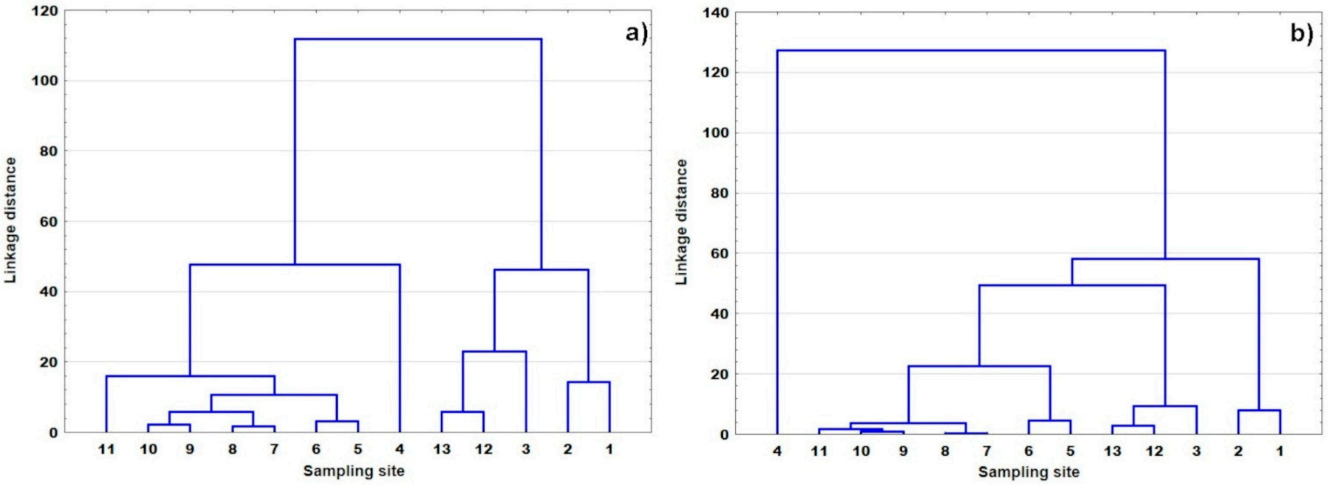

Figure S1. Dendrograms depicting the clustering of sampling sites in (a) 2018 and (b) 2019 for temperature.

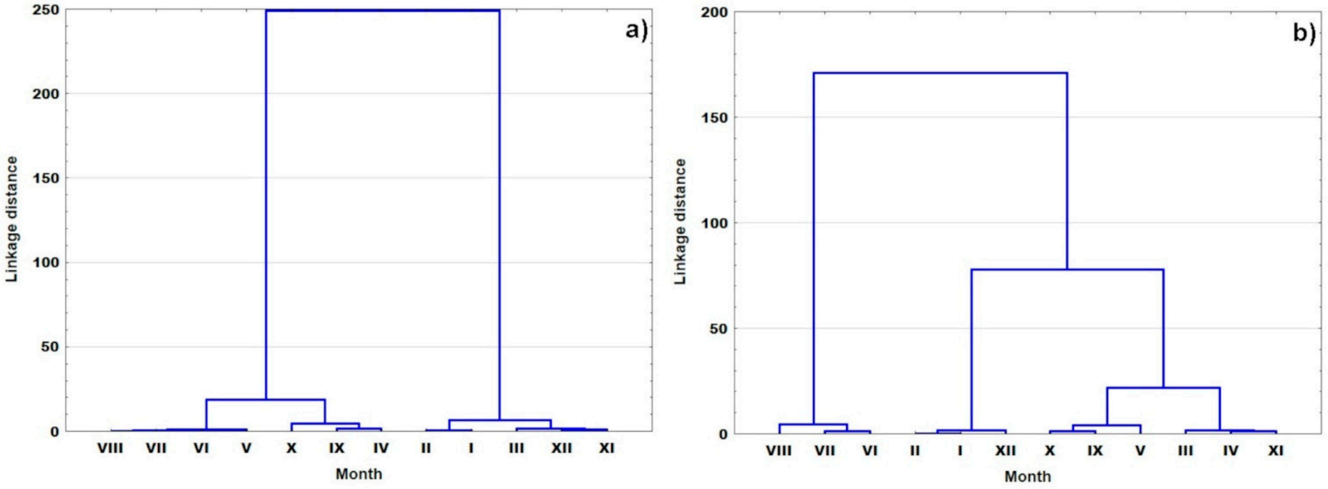

Figure S2. Dendrograms depicting the clustering of months in (a) 2018 and (b) 2019 for temperature.

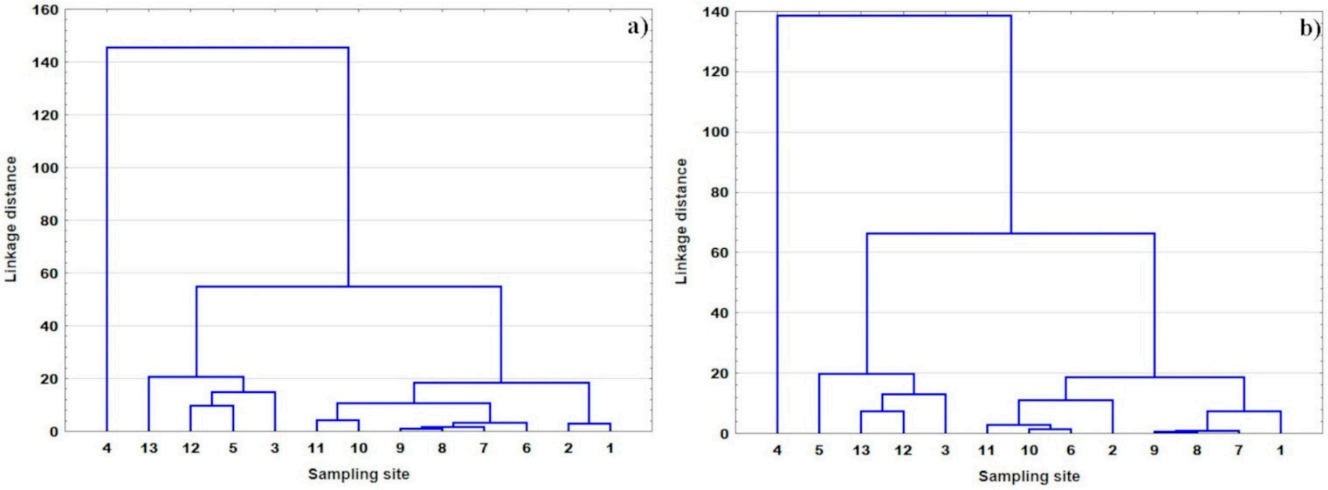

Figure S3. Dendrograms depicting the clustering of sampling sites in (a) 2018 and (b) 2019 for dissolved oxygen.

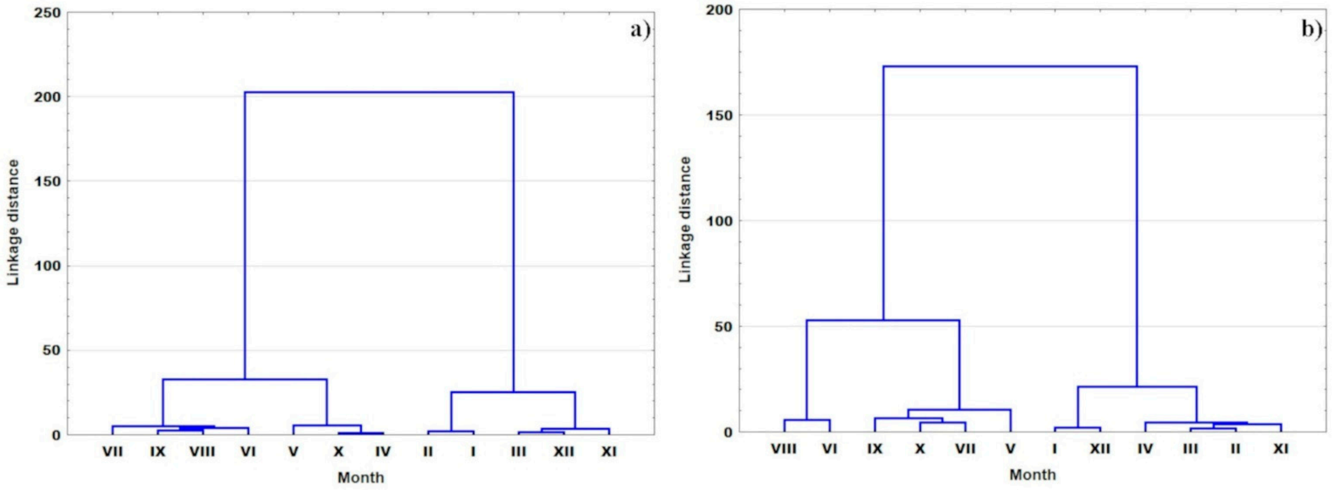

Figure S4. Dendrograms depicting the clustering of months in (a) 2018 and (b) 2019 for dissolved oxygen.

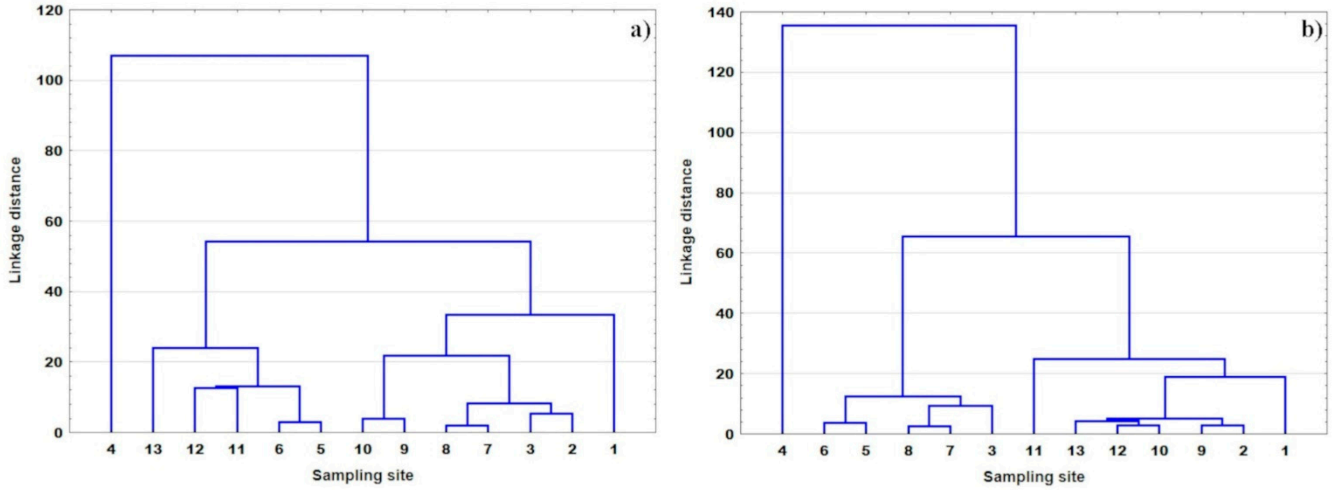

Figure S5. Dendrograms depicting the clustering of sampling sites in (a) 2018 and (b) 2019 for DOC.

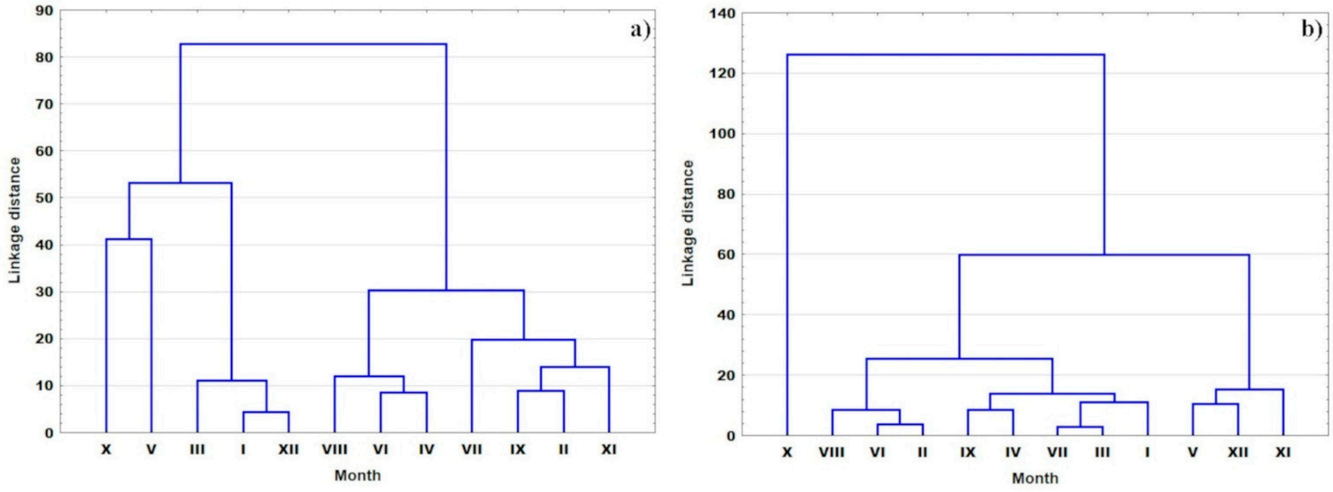

Figure S6. Dendrograms depicting the clustering of months in (a) 2018 and (b) 2019 for DOC.

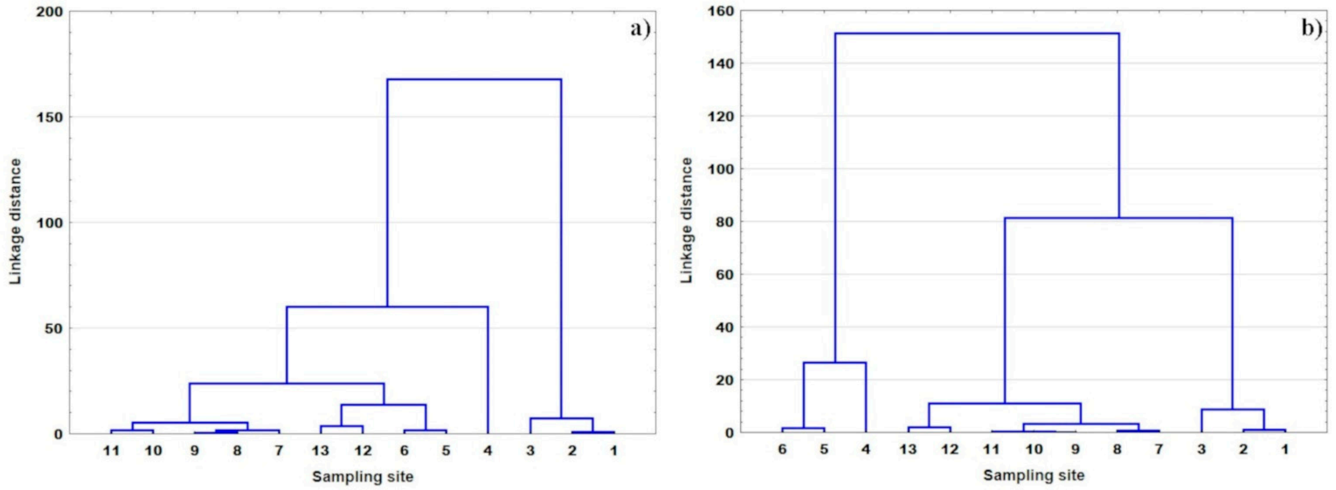

Figure S7. Dendrograms depicting the clustering of sampling sites in (a) 2018 and (b) 2019 for electrical conductivity.

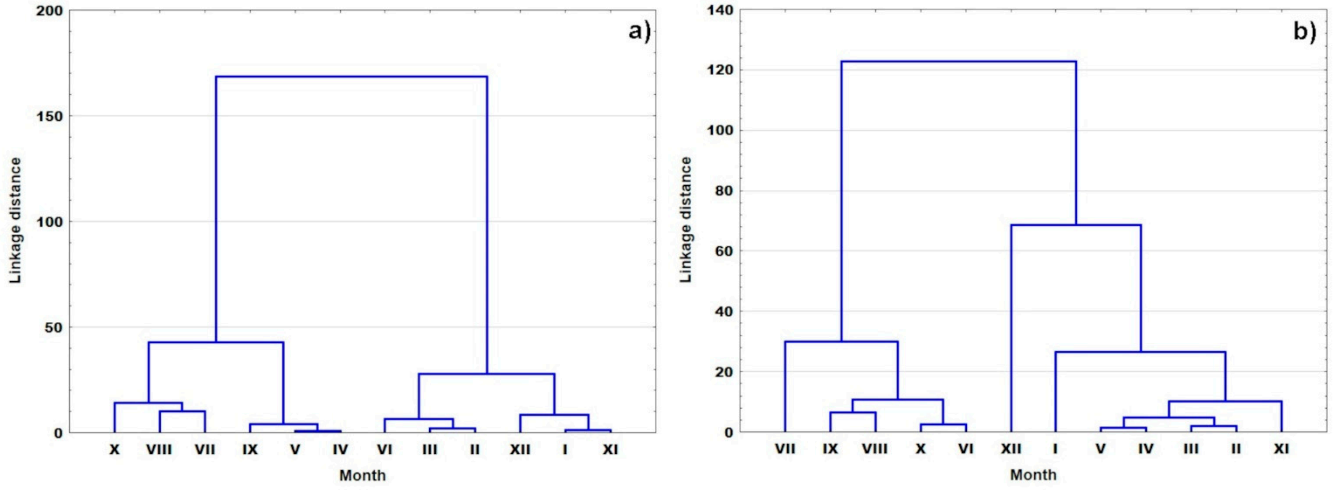

Figure S8. Dendrograms depicting the clustering of months in (a) 2018 and (b) 2019 for electrical conductivity.

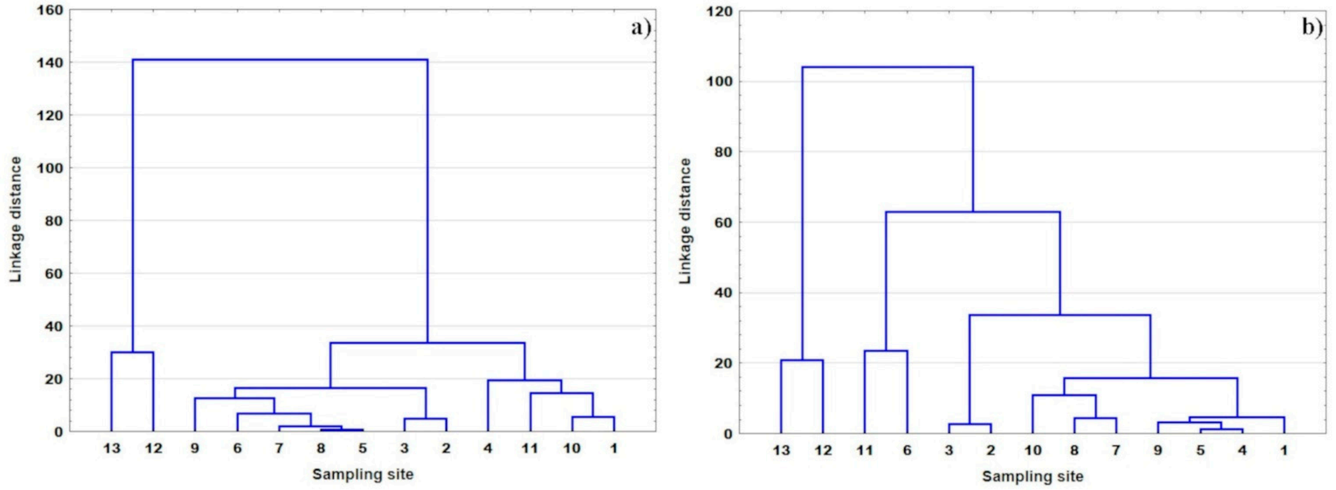

Figure S9. Dendrograms depicting the clustering of sampling sites in (a) 2018 and (b) 2019 for nitrates.

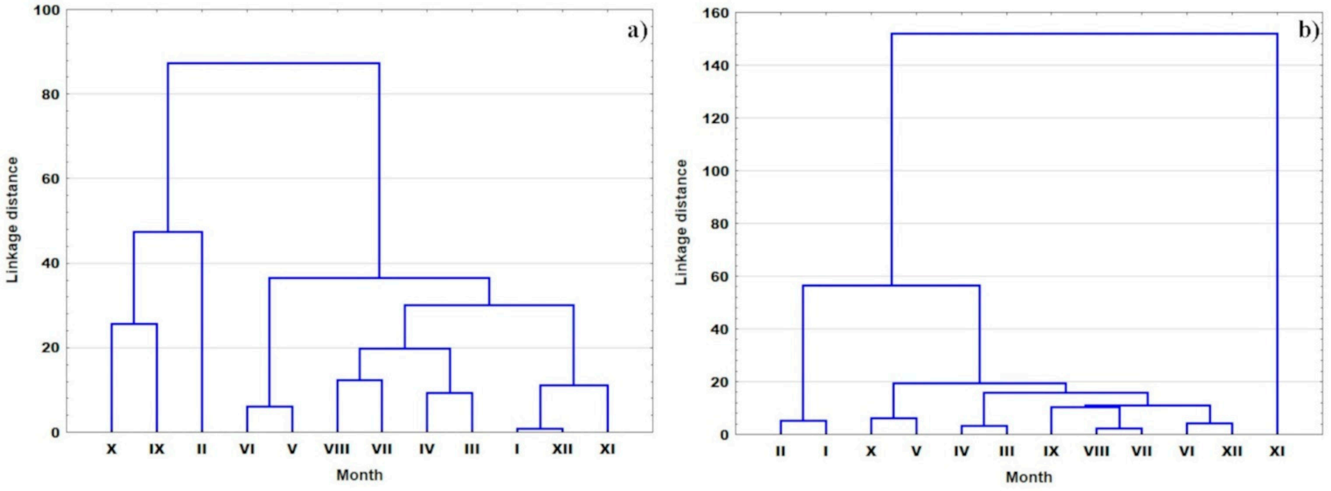

Figure S10. Dendrograms depicting the clustering of months in (a) 2018 and (b) 2019 for nitrates.

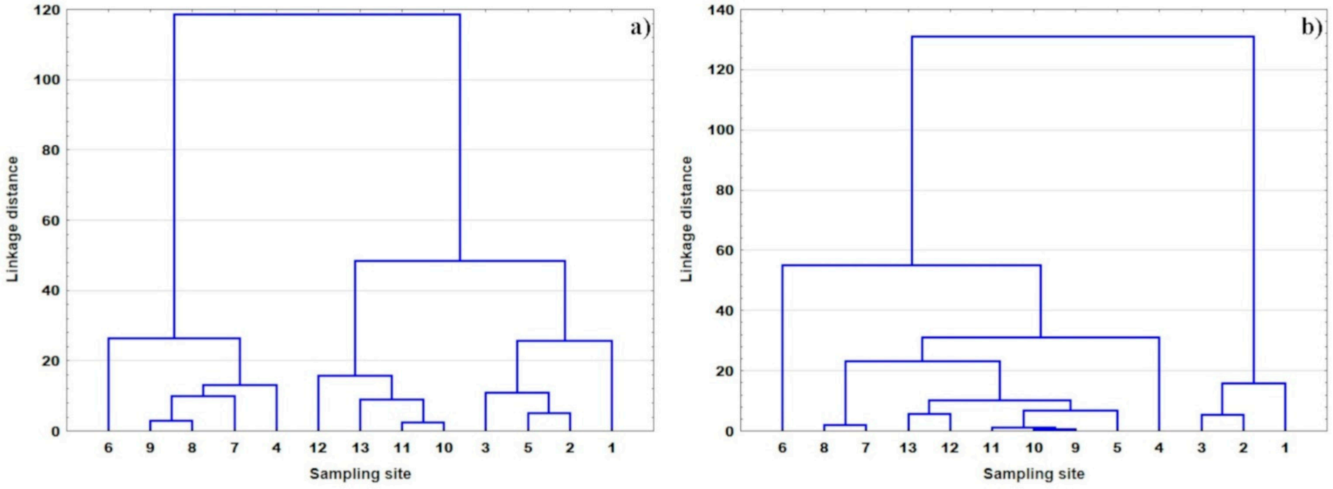

Figure S11. Dendrograms depicting the clustering of sampling sites in (a) 2018 and (b) 2019 for phosphates.

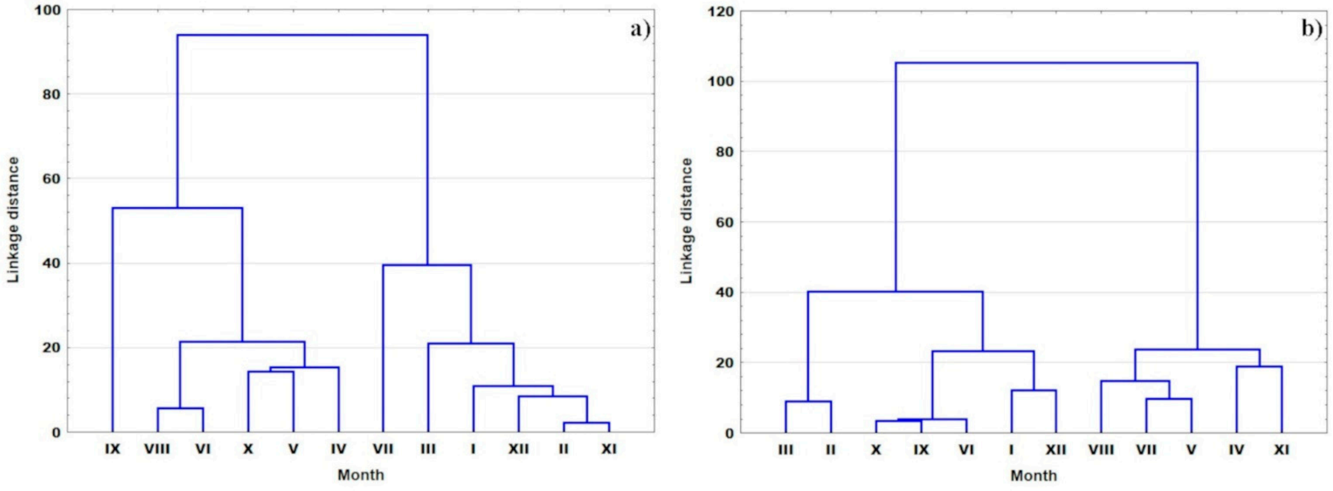

Figure S12. Dendrograms depicting the clustering of months in (a) 2018 and (b) 2019 for phosphates.

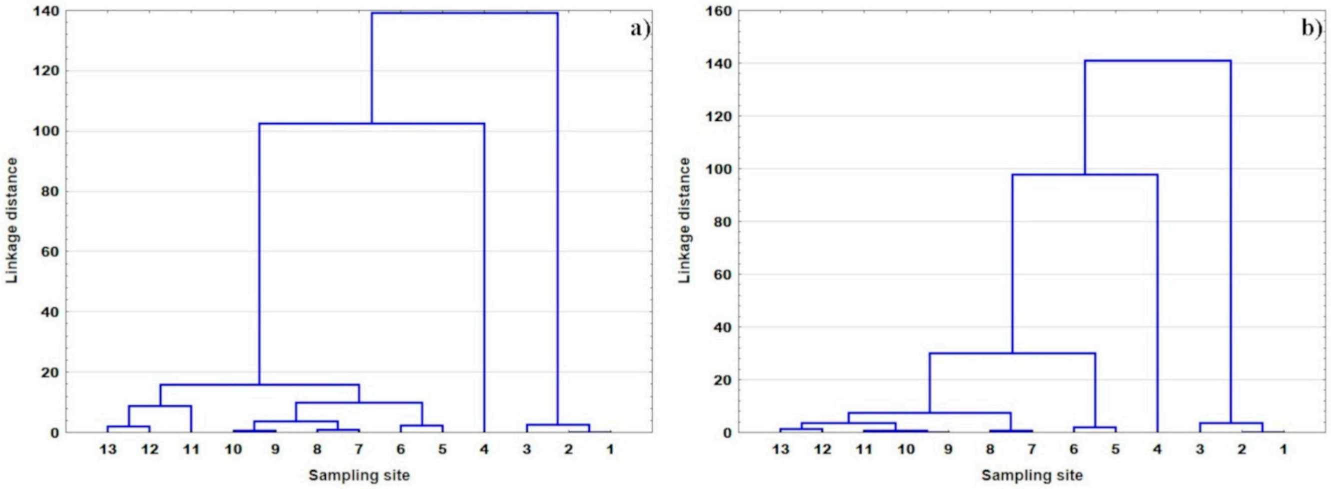

Figure S13. Dendrograms depicting the clustering of sampling sites in (a) 2018 and (b) 2019 for sodium.

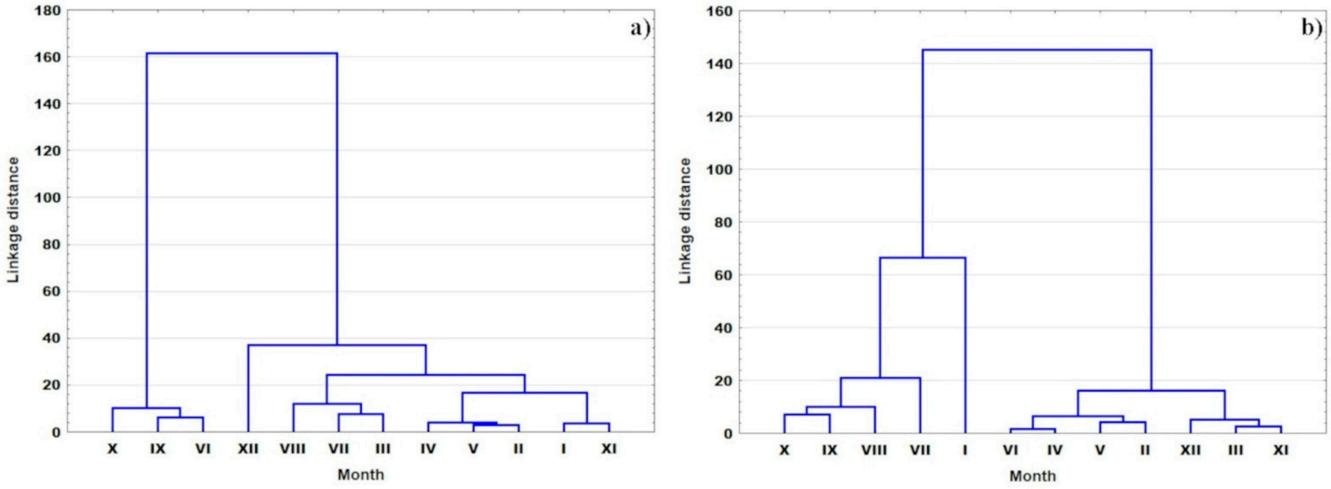

Figure S14. Dendrograms depicting the clustering of months in (a) 2018 and (b) 2019 for sodium.

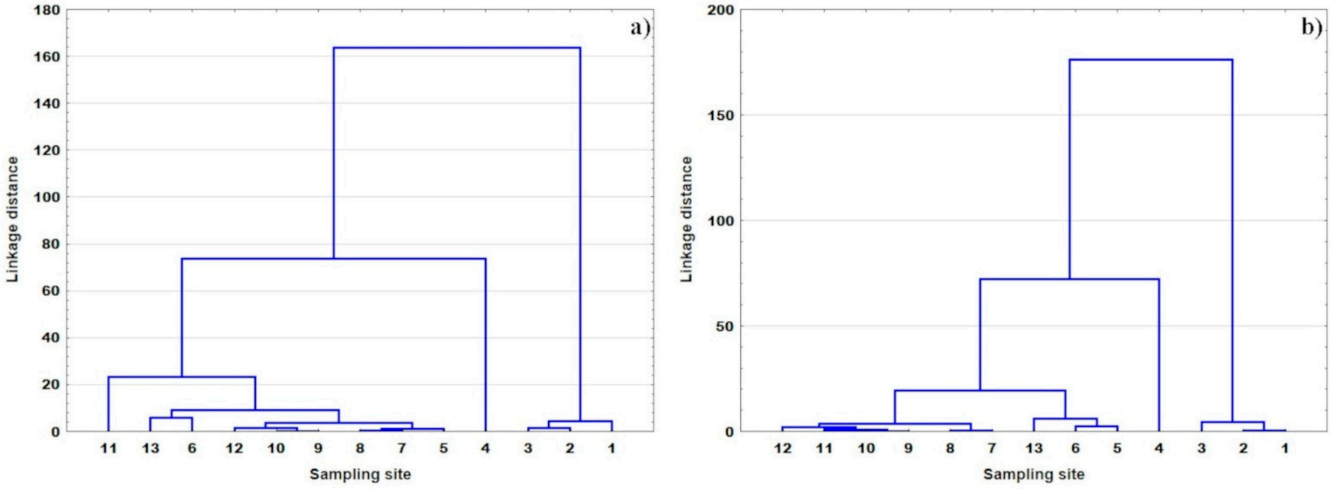

Figure S15. Dendrograms depicting the clustering of sampling sites in (a) 2018 and (b) 2019 for potassium.

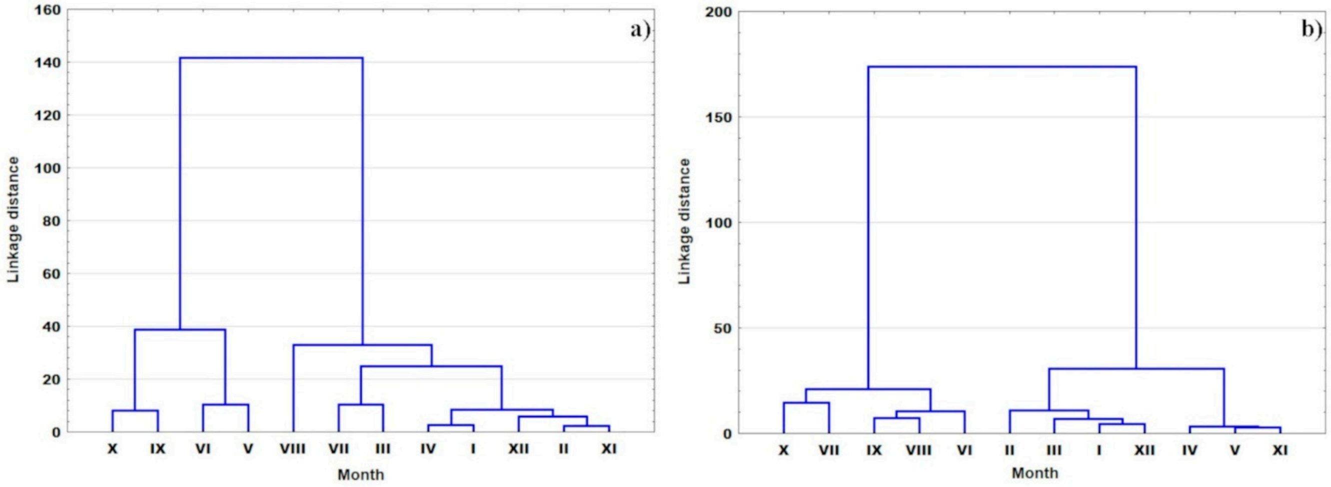

Figure S16. Dendrograms depicting the clustering of months in (a) 2018 and (b) 2019 for potassium.

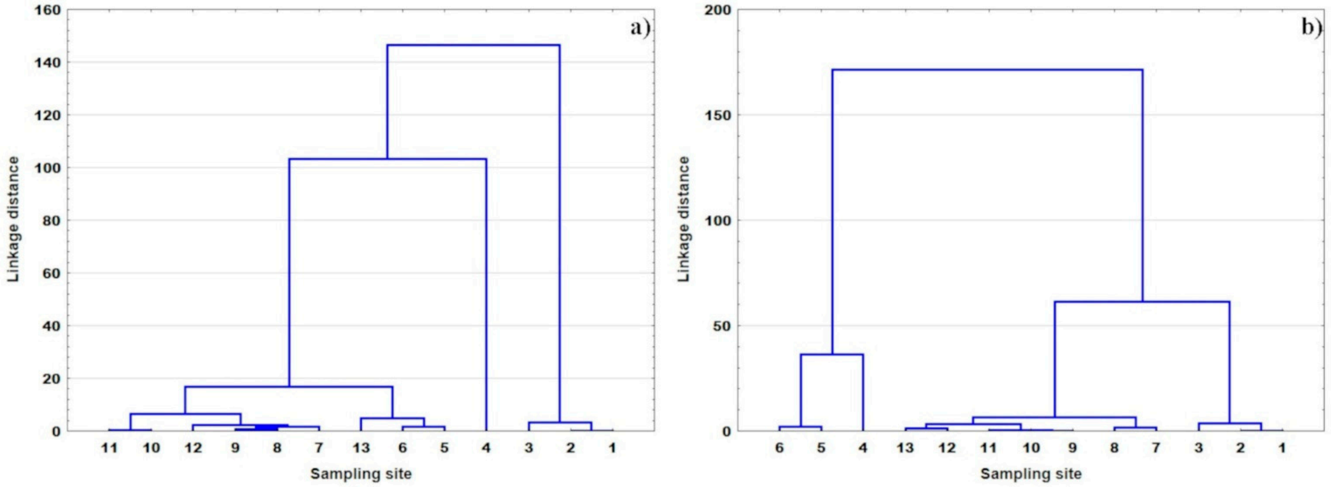

Figure S17. Dendrograms depicting the clustering of sampling sites in (a) 2018 and (b) 2019 for chlorides.

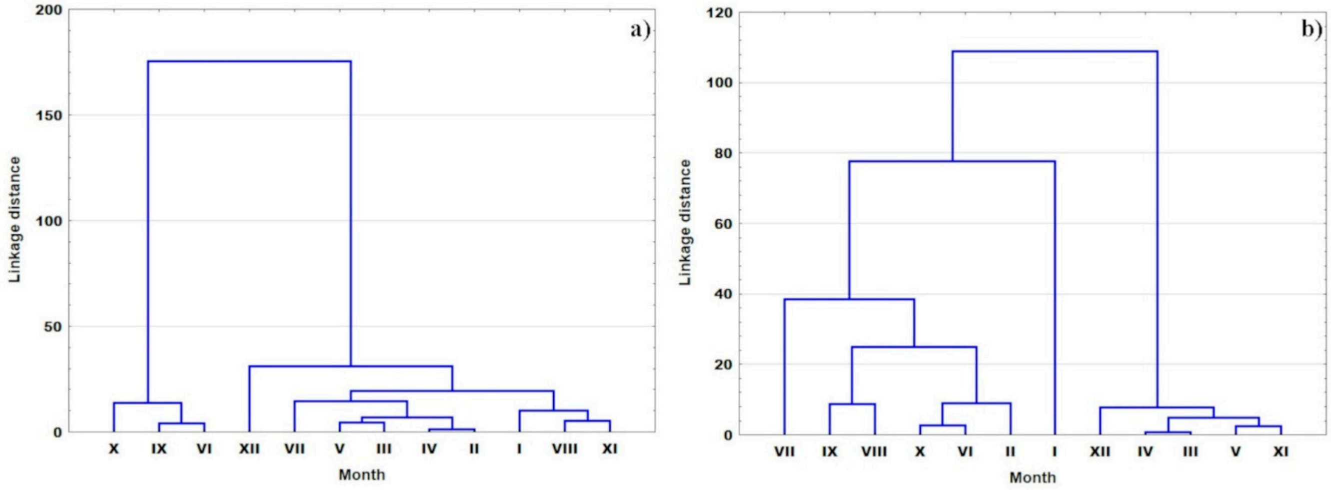

Figure S18. Dendrograms depicting the clustering of months in (a) 2018 and (b) 2019 for chlorides.

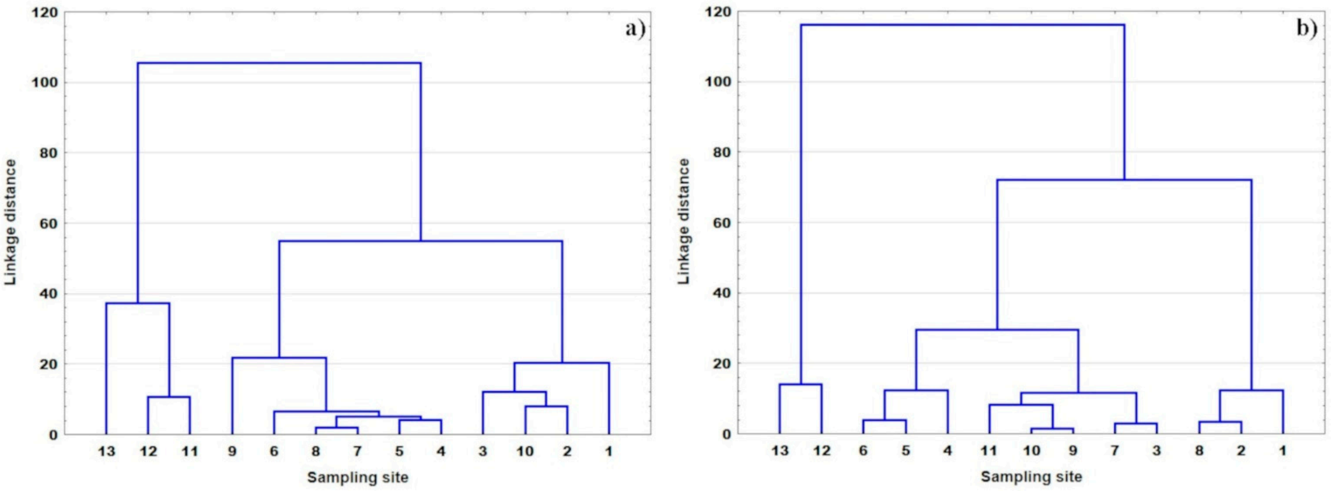

Figure S19. Dendrograms depicting the clustering of sampling sites in (a) 2018 and (b) 2019 for calcium.

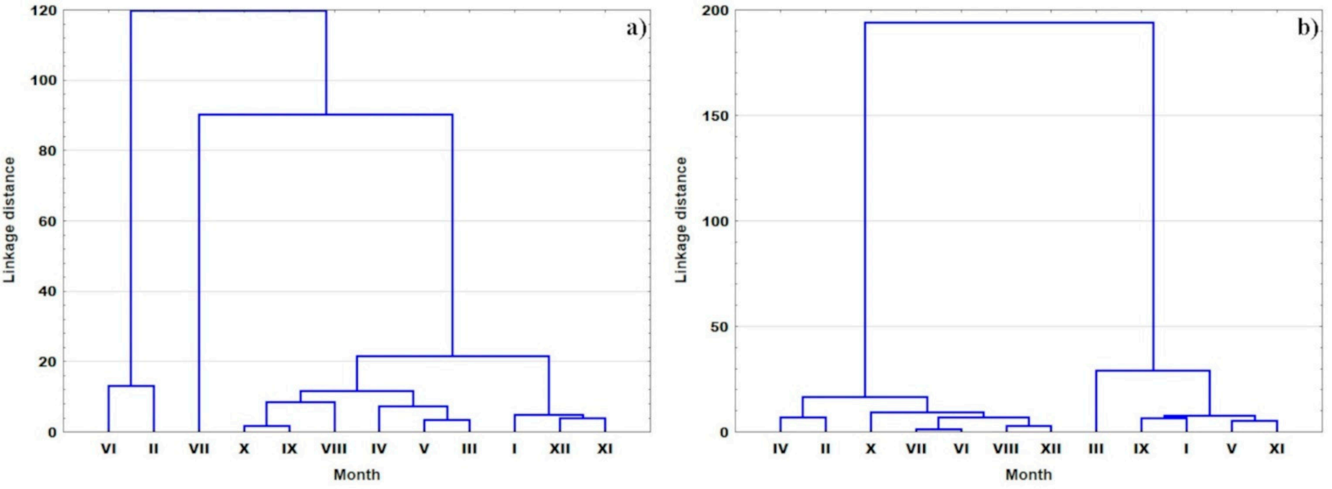

Figure S20. Dendrograms depicting the clustering of months in (a) 2018 and (b) 2019 for calcium.

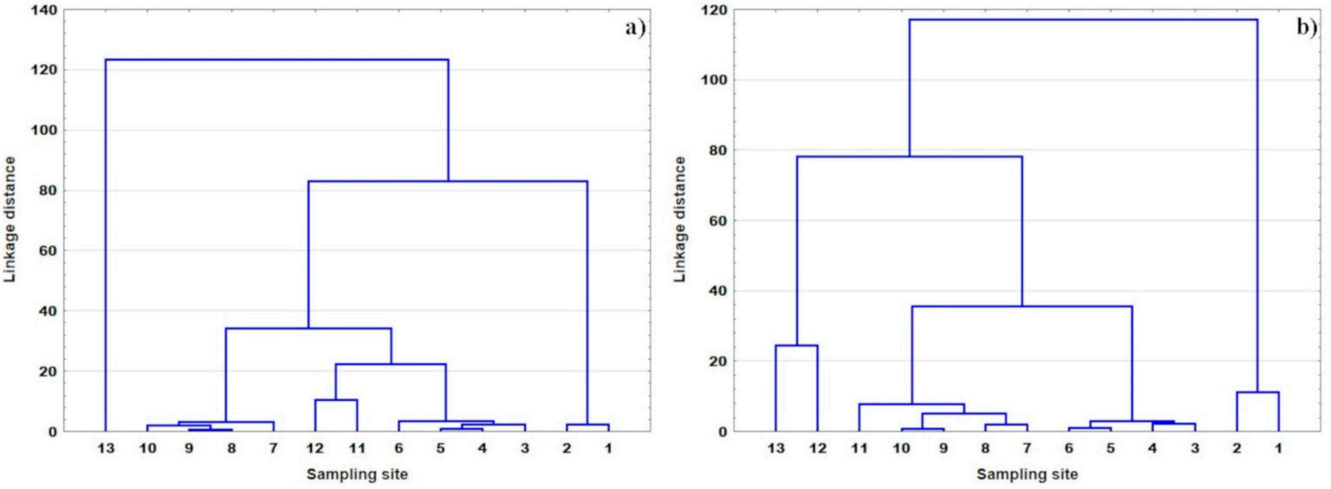

Figure S21. Dendrograms depicting the clustering of sampling sites in (a) 2018 and (b) 2019 for magnesium.

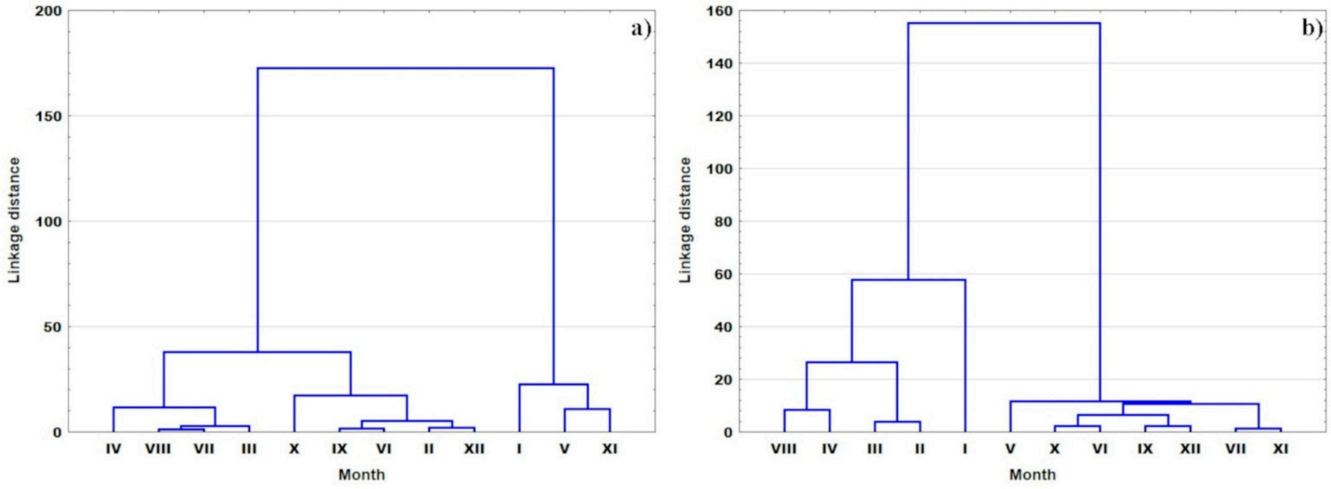

Figure S22. Dendrograms depicting the clustering of months in (a) 2018 and (b) 2019 for magnesium.

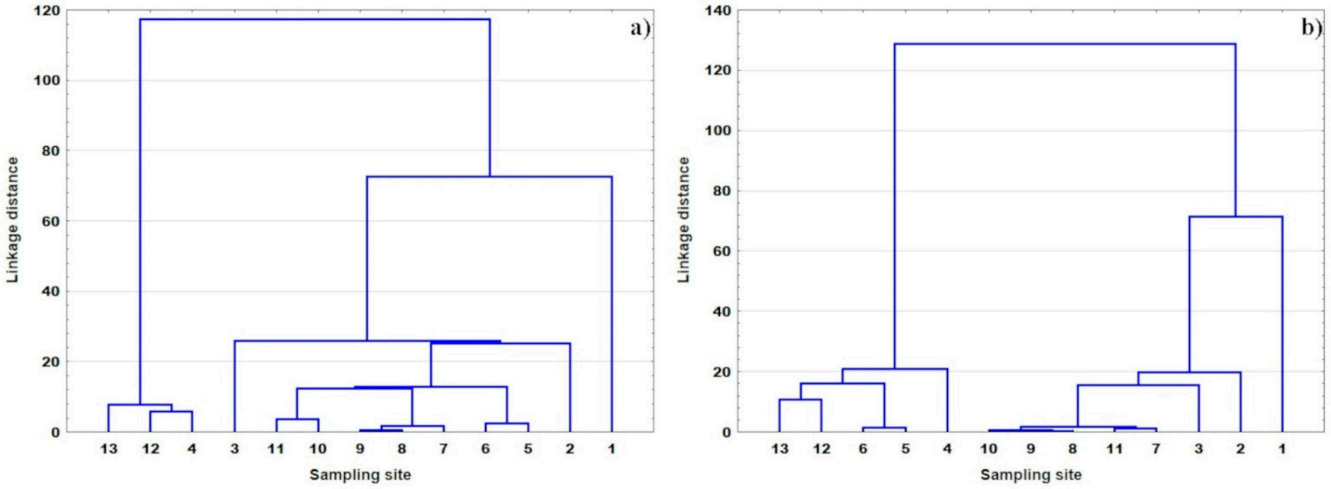

Figure S23. Dendrograms depicting the clustering of sampling sites in (a) 2018 and (b) 2019 for bicarbonates.

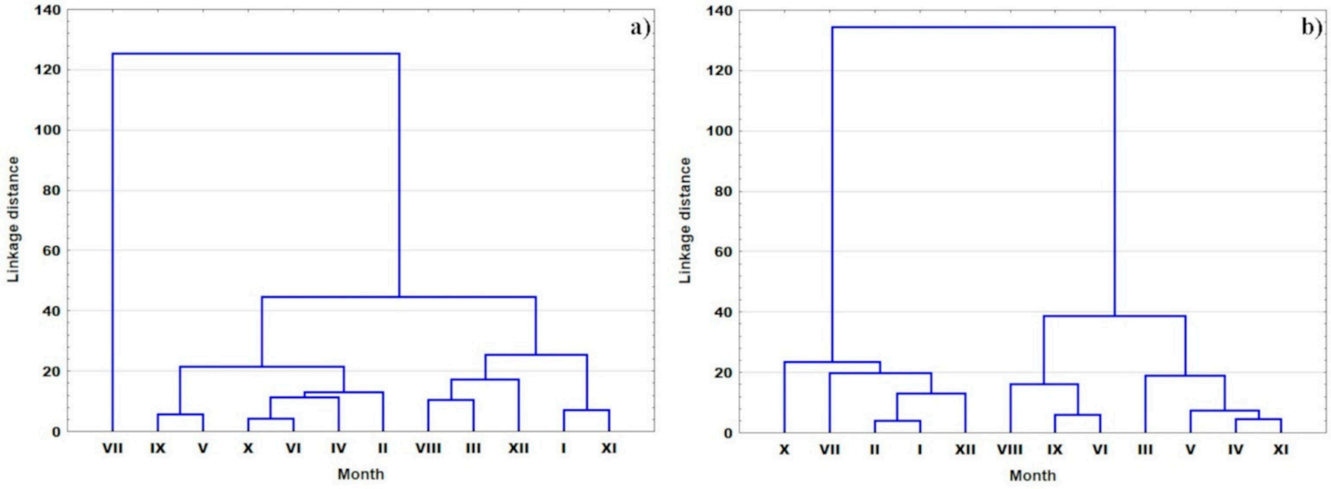

Figure S24. Dendrograms depicting the clustering of months in (a) 2018 and (b) 2019 for bicarbonates.

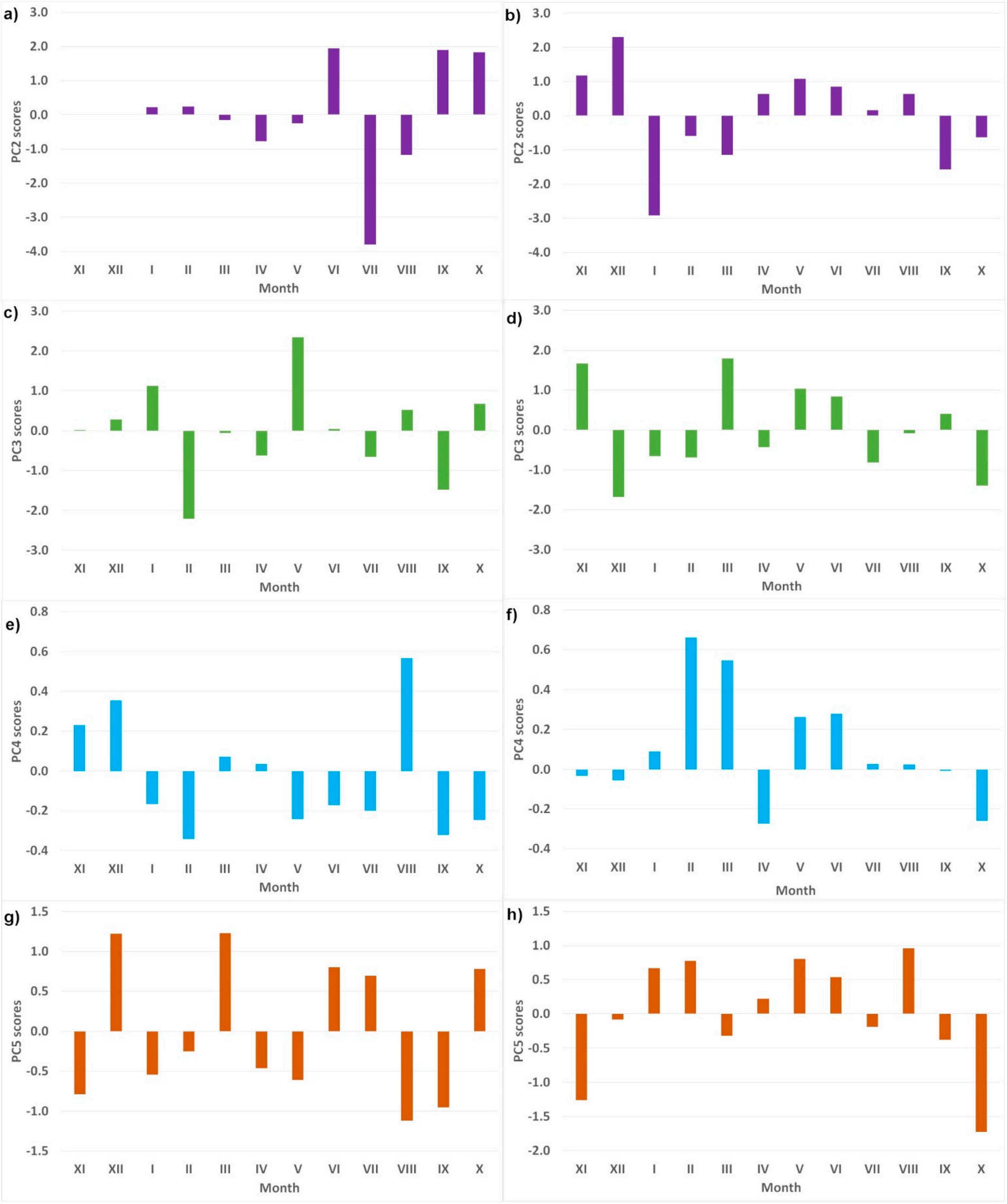

Figure S25. Score values of PC2, PC3, PC4, and PC5 in (a,b,c,d) 2018 and (e,f,g,h) 2019 for the individual months.

Table S9. Factor loadings of variables calculated based on the monthly average values of the determined parameters for 2018 and 2019.

	2018					2019				
	**PC1**	**PC2**	**PC3**	**PC4**	**PC5**	**PC1**	**PC2**	**PC3**	**PC4**	**PC5**
Eigenvalue	**5.3**	**2.5**	**1.4**	**0.9**	**0.8**	**5.9**	**2.0**	**1.3**	**1.0**	**0.7**
% of the total variance	**44.3**	**20.7**	**11.7**	**7.9**	**6.4**	**49.6**	**17.1**	**11.0**	**8.6**	**5.9**
EC	**-0.74**	0.38	-0.10	0.35	0.22	**0.85**	-0.47	0.13	-0.05	0.14
temp	**0.91**	-0.14	0.18	0.23	-0.15	**0.93**	0.25	0.12	-0.03	-0.07
DO	**-0.93**	0.14	-0.10	-0.17	0.07	**-0.90**	-0.28	-0.23	0.09	-0.14
DOC	-0.47	0.08	**0.78**	-0.25	0.16	0.37	*-0.52*	-0.43	-0.26	*-0.53*
NO_3	*-0.53*	0.10	*-0.69*	-0.34	-0.29	-0.46	-0.17	0.37	*0.66*	-0.25
PO_4	**0.74**	0.34	0.08	0.07	-0.47	*0.58*	0.47	-0.13	*0.55*	0.03
HCO_3	-0.48	**0.79**	0.06	0.03	-0.30	*0.61*	-0.08	*0.64*	-0.27	0.03
Cl	*0.62*	*0.65*	-0.20	-0.24	0.28	*0.65*	*-0.62*	-0.08	0.26	0.25
Na	*0.66*	*0.60*	-0.13	-0.17	0.38	**0.78**	-0.43	-0.26	0.28	-0.06
K	**0.77**	0.45	0.11	-0.20	-0.06	**0.97**	-0.01	0.06	0.03	-0.10
Ca	-0.32	*0.68*	0.03	*0.57*	-0.01	-0.40	-0.44	*0.64*	0.02	-0.24
Mg	*-0.51*	0.33	0.43	-0.32	-0.23	*-0.60*	*-0.63*	-0.06	-0.01	0.43

Table S10. WQI values and water quality assessment [27] for individual months and measurement points in 2018 and 2019.

Date	Site 1	2	3	4	5	6	7	8	9	10	11	12	13	14	15	16	17
**XI.2017**	53	82	85	177	112	125	148	150	154	125	131	162	121				
**XII.2017**	17	46	56	143	93	151	266	262	208	140	110	133	110				
**I.2018**	55	92	114	132	119	286	258	125	136	116	108	141	109				
**II.2018**	34	70	84	157	97	150	117	124	130	124	128	105	111				
**III.2018**	55	80	52	150	82	210	130	99	90	98	120	137	168				
**IV.2018**	37	103	73	299	242	291	264	242	225	181	78	170	121				
**V.2018**	63	148	18	296	136	**408**	286	256	194	159	166	99	131				
**VI.2018**	83	173	227	**305**	215	**315**	223	223	**322**	228	182	151	137				
**VII.2018**	101	121	230	188	129	168	169	171	143	**308**	**328**	205	215				
**VIII.2018**	130	176	237	234	178	277	286	277	258	224	219	145	182				
**IX.2018**	**300**	100	162	159	153	**342**	**361**	287	236	217	246	**404**	265				
**X.2018**	31	65	74	161	138	**318**	248	201	201	166	161	159					
**XI.2018**	79	192	112	163	163	**327**	**318**	288	249	180	234	**308**	233	140	144	151	137
**XII.2018**	57	81	77	143	94	**377**	135	124	135	144	130	174	164	183	128	158	203
**I.2019**	64	22	87	132	88	**356**	253	225	150	134	99	92	80	122	117	81	115
**II.2019**	72	88	78	179	140	112	141	140	155	155	163	157	146	73	60	63	55
**III.2019**	2	17	22	121	74	75	101	86	74	74	70	70	27	49	38	28	35
**IV.2019**	22	124	43	192	116	**384**	257	297	211	191	171	104	86	61	66	61	57
**V.2019**	46	113	129	243	154	195	244	224	215	228	190	146	218	106	95	95	119
**VI.2019**	51	108	152	**335**	225	**301**	197	189	187	167	134	124	159	136	115	111	118
**VII.2019**	54	103	139	295	241	**408**	263	242	225	221	214	242	164	69	52	43	47
**VIII.2019**	145	196	298	**338**	286	**329**	**356**	**310**	206	201	199	180	174	141	135	125	93
**IX.2019**	62	107	147	233	152	273	187	185	161	190	156	137	144	79	77	67	78
**X.2019**	74	125	143	253	209	220	181	135	185	181	155	146	154	81	101	80	60

excellent; good; poor; very poor; unsuitable
